# Breathing can be dangerous: Opportunistic fungal pathogens and the diverse community of the small mammal lung mycobiome

**DOI:** 10.3389/ffunb.2022.996574

**Published:** 2022-09-26

**Authors:** Paris S. Salazar-Hamm, Kyana N. Montoya, Liliam Montoya, Kel Cook, Schuyler Liphardt, John W. Taylor, Joseph A. Cook, Donald O. Natvig

**Affiliations:** ^1^ Department of Biology, University of New Mexico, Albuquerque, NM, United States; ^2^ Department of Plant and Microbial Biology, University of California, Berkeley, Berkeley, CA, United States; ^3^ Museum of Southwestern Biology, University of New Mexico, Albuquerque, NM, United States

**Keywords:** *Coccidioides*, Valley fever, Onygenales, *Blastomyces*, *Aspergillus fumigatus*, *Pneumocystis*

## Abstract

Human lung mycobiome studies typically sample bronchoalveolar lavage or sputum, potentially overlooking fungi embedded in tissues. Employing ultra-frozen lung tissues from biorepositories, we obtained fungal ribosomal RNA ITS2 sequences from 199 small mammals across 39 species. We documented diverse fungi, including common environmental fungi such as *Penicillium* and *Aspergillus*, associates of the human mycobiome such as *Malassezia* and *Candida*, and others specifically adapted for lungs (*Coccidioides*, *Blastomyces*, and *Pneumocystis*). *Pneumocystis* sequences were detected in 83% of the samples and generally exhibited phylogenetic congruence with hosts. Among sequences from diverse opportunistic pathogens in the Onygenales, species of *Coccidioides* occurred in 12% of samples and species of *Blastomyces* in 85% of samples. *Coccidioides* sequences occurred in 14 mammalian species. The presence of neither *Coccidioides* nor *Aspergillus fumigatus* correlated with substantial shifts in the overall mycobiome, although there was some indication that fungal communities might be influenced by high levels of *A. fumigatus*. Although members of the Onygenales were common in lung samples (92%), they are not common in environmental surveys. Our results indicate that *Pneumocystis* and certain Onygenales are common commensal members of the lung mycobiome. These results provide new insights into the biology of lung-inhabiting fungi and flag small mammals as potential reservoirs for emerging fungal pathogens.

## Introduction

Human lungs are estimated to inhale 500 to 100,000 fungal spores per day depending on local environmental conditions (Hasleton, 1972; Hamm et al., 2020b). Substantial attention has been paid to mechanisms fungi use to avoid the immune response that lungs can mount against pathogens (e.g. Kwon-Chung and Sugui, 2013; Wiesner and Klein, 2017; Ward et al., 2021). Only recently, however, has it been suggested that healthy lungs may harbor as many fungi as lungs diagnosed with overt infections (Richardson et al., 2019), but it remains unclear which fungi in healthy lung tissues present transiently as a result of spore inhalations and which fungi survive as commensals, pathogens, or mutualists.

Once thought to represent a sterile tissue, the lung is now known to possess a microbiome, including a fungal component, the mycobiome (Hamm et al., 2020b). Studies of the human lung mycobiome have been limited, with samples typically derived from sputum or bronchoalveolar lavage rather than from actual tissues. Previous studies typically focused on diseased lungs (Nguyen et al., 2015; Weaver et al., 2019), so knowledge of the human lung mycobiome and more broadly the mammalian lung mycobiome remains limited.

Museum collections provide opportunities to study animal-microbe interactions across broad spatial scales and temporal archives (Cheng et al., 2011; Carvalho et al., 2017; Dunnum et al., 2017; Schindel and Cook, 2018). The goal of the current study was to explore the value of ultra-frozen lung tissues from museum collections to characterize the lung mycobiome of wild small mammals using molecular and cultivation methods. Lung samples were chosen to represent the species diversity from arid environments across the southwestern United States, in part to determine whether such frozen tissues would help define the range and host species of *Coccidioides*, the causative agent of coccidioidomycosis (Valley fever).

Long before the advent of modern molecular methods there were indications in the 1940s and 1950s that apparently healthy mammals frequently harbor living fungi in their lung tissues. The evidence for this arose in part from attempts by Dr. C. W. Emmons (Emmons and Ashburn, 1942; Emmons, 1943) to determine whether rodents serve as natural reservoirs for coccidioidomycosis. Those studies demonstrated a relatively high incidence of *Coccidioides* among specific rodent hosts at certain localities (15% of pocket mice and 17% kangaroo rats at San Carlos Indian Reservation, San Carlos, Arizona; Emmons and Ashburn, 1942). Moreover, these and other studies demonstrated that a related fungus, now generally known as *Blastomyces parvus* (previously *Haplosporangium parvum* or *Emmonsia parva*), was detected even more frequently in mammalian lung tissues of a wide variety of mammalian hosts (Emmons and Ashburn, 1942; Jellison, 1950; Bakerspigel, 1956).

Species of *Pneumocystis* represent a special case in the context of the lung mycobiome, because they are obligately symbiotic lung fungi. They are broadly distributed across mammals, and it has been suggested that each mammalian species has at least one host-specific species of *Pneumocystis* (Danesi et al., 2020). Molecular surveys for *Pneumocystis* in domesticated animals and wildlife have used targeted PCR approaches, and success rates have been mixed depending on the animal species targeted (Demanche et al., 2001; Akbar et al., 2012; Danesi et al., 2020). We report that high-throughput sequencing targeting fungal ribosomal internal transcribed spacer (ITS) sequences identifies this group at high frequency (83%) among diverse and widely distributed small mammals. This approach also provides preliminary information regarding specific fungal-host associations and coevolutionary histories for this group.

Our molecular study also expands the results of early lung studies with respect to *Coccidioides* and *Blastomyces* and demonstrates that mammalian lung tissues host a diverse mycobiome, with many species either known or now appearing to be adapted to the lung environment. Our results further indicate that, along with Pneumocystidales, members of the Onygenales, Malasseziales, and Saccharomycetales are common constituents of the natural lung mycobiome. We hypothesize that members of all four orders are typically benign commensals rather than aggressive opportunistic pathogens and that the lung mycobiome provides an excellent opportunity to better understand the evolution of commensalism or pathogenicity.

## Results

### Tissue acquisition

A total of 199 ultra-frozen lung tissues were obtained through formal request from the University of New Mexico Museum of Southwestern Biology (MSB) and the University of California Berkeley Museum of Vertebrate Zoology (MVZ). Samples represented 39 species from six mammalian families (Heteromyidae, Cricetidae, Muridae, Sciuridae, Geomyidae, and Leporidae) (**Supplementary Table 1**). Samples spanned 45 localities within 19 counties across California, Arizona, and New Mexico. Museum specimen collection dates ranged from 1994 to 2019.

### Confirmation of host identities

Because species designations that accompany most museum specimens are initially morphology based, we sequenced a region of the mitochondrial cytochrome b (cyt b) gene to test mammalian host identities. We successfully captured mitochondrial cyt b sequences, a common molecular barcode, from 156 of 199 samples to confirm or correct host identifications. We found that 22 of the frozen tissue samples had incorrect initial species designations. The analyses presented here reflect species designations based on cyt b sequences (**Supplementary Table 1**).

### Lung mycobiome community analysis

**Figure 1:**
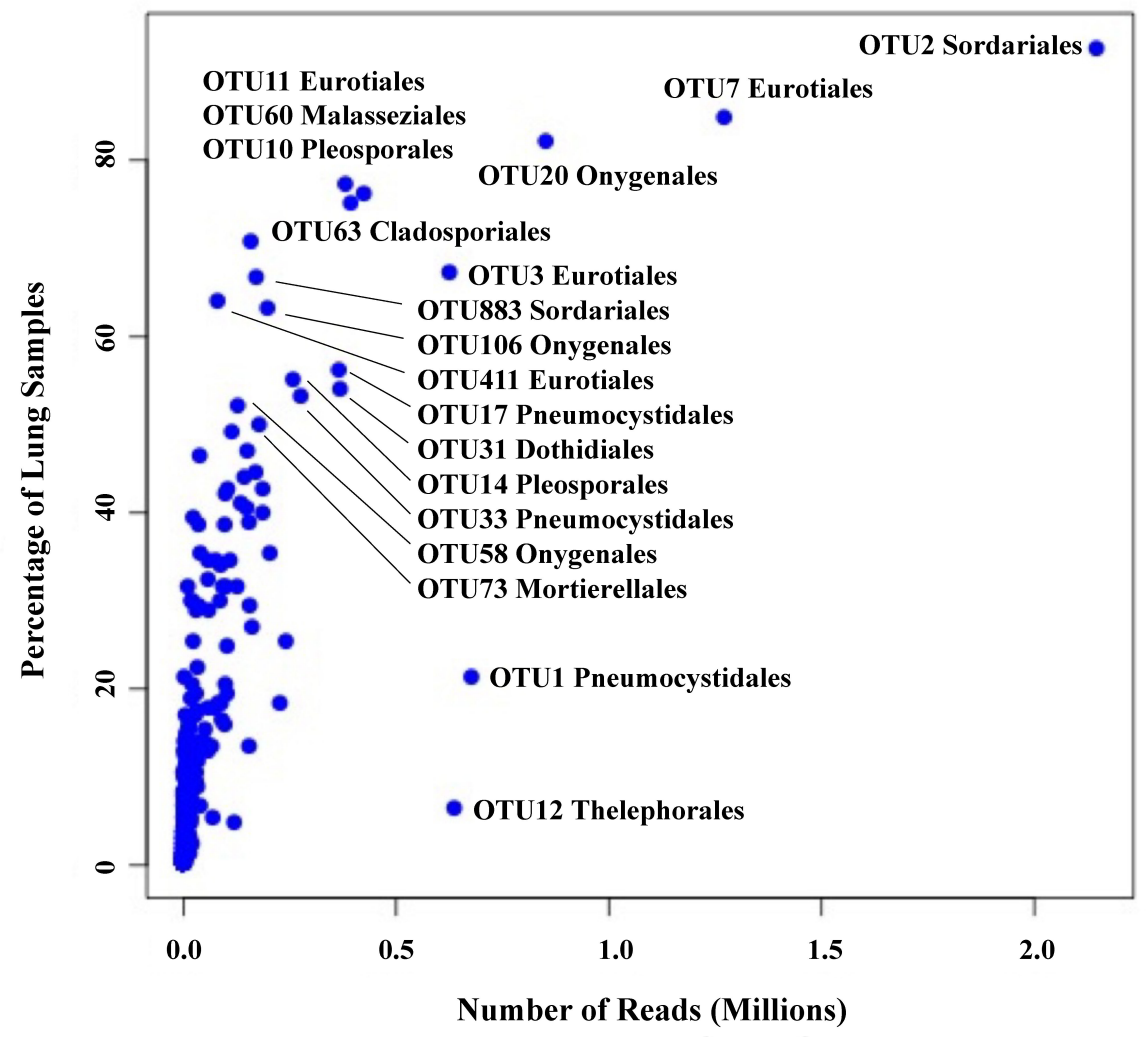
Relationship between frequency (percent of lung samples) and total reads for 762 OTUs. Taxonomic orders are given for those OTUs with a frequency of ≥50% plus two OTUs with high read counts, OTU1 (Pneumocystidales) and OTU12 (Thelephorales).

We employed Illumina sequencing that targeted the fungal nuclear ribosomal RNA ITS2 region for fungal community analyses. Processing of ITS2 sequences with UPARSE of 199 small-mammalian lung samples produced a total of 16,515,699 sequences clustered into 762 operational taxonomic units (OTUs; Blaxter et al., 2005). The average number of fungal OTUs per sample was 43.7 (**Supplementary Data Sheet 2**). Ascomycota (48%) and Basidiomycota (20%) were the dominant phyla with only a few OTUs from Mucoromycota (3%). Twenty-nine percent of the OTUs could not be identified to phylum using either NCBI or UNITE databases. Among the most abundant and frequent OTUs were members of the Eurotiomycetes (*Aspergillus*, *Penicillium*, and *Blastomyces*), Sordariomycetes (Sordariaceae), Pneumocystidomycetes (*Pneumocystis*), Dothideomycetes (*Alternaria, Curvularia*, and *Aureobasidium*), Saccharomycetes (*Candida* and *Geotrichum*) and Malasseziomycetes (*Malassezia*) (**Table 1**). There was a strong correlation between OTU frequency (percentage of samples with a given OTU) and abundance (sequence read numbers for a given OTU) (**Figure 1**).

**Table 1:** Prevalence of major fungal taxa from ITS2 Illumina sequencing of 199 small-mammal lung samples.

Phylum	Class	Order (total OTUs)	Genera	Prevalence
Ascomycota	Eurotiomycetes	Eurotiales (36)		99%
			*Penicillium*	93%
			*Aspergillus*	93%
			*Thermomyces*	42%
			*Rasamsonia*	19%
		Onygenales (19)		92%
			*Blastomyces Emmonsiellopsis*	85% 22%
			*Auxarthron*	20%
			*Emmonsia*	16%
			*Coccidioides*	12%
	Sordariomycetes	Sordariales (20)		98%
			Sordariaceae^a^	94%
			*Botryotrichum*	34%
			*Canariomyces*	32%
	Pneumocystidomycetes	Pneumocystidales (21)	*Pneumocystis*	83%
	Dothideomycetes	Pleosporales (45)		98%
			*Alternaria*	86%
			*Curvularia*	54%
			*Herpotrichia*	32%
			*Phoma*	30%
			*Preussia*	22%
		Cladosporiales (2)	*Cladosporium*	71%
		Dothideales (8)		68%
			*Aureobasidium*	55%
			*Kabatiella*	27%
	Saccharomycetes	Saccharomycetales (13)		75%
			*Candida*	49%
			*Geotrichum*	47%
			*Cyberlindnera*	35%
			*Clavispora*	18%
			*Debaryomyces*	12%
Basidiomycota	Malasseziomycetes	Malasseziales (8)	*Malassezia*	83%
	Tremellomycetes	Filobasidiales		46%
			*Naganishia*	22%
			*Filobasidium*	15%
			*Solicoccozyma*	14%

OTUs of the same genus were combined to determine the percentage of samples in which they were observed, and those in greater than 10% of the samples are displayed.

a Combined result for OTU2 (93% of the samples), which had a highest BLAST hit to an uncultured Sordariaceae sequence and second highest hit to *Neurospora* spp., and OTU197 (35% of the samples), which had a highest hit to *Neurospora* spp.

**Figure 2:**
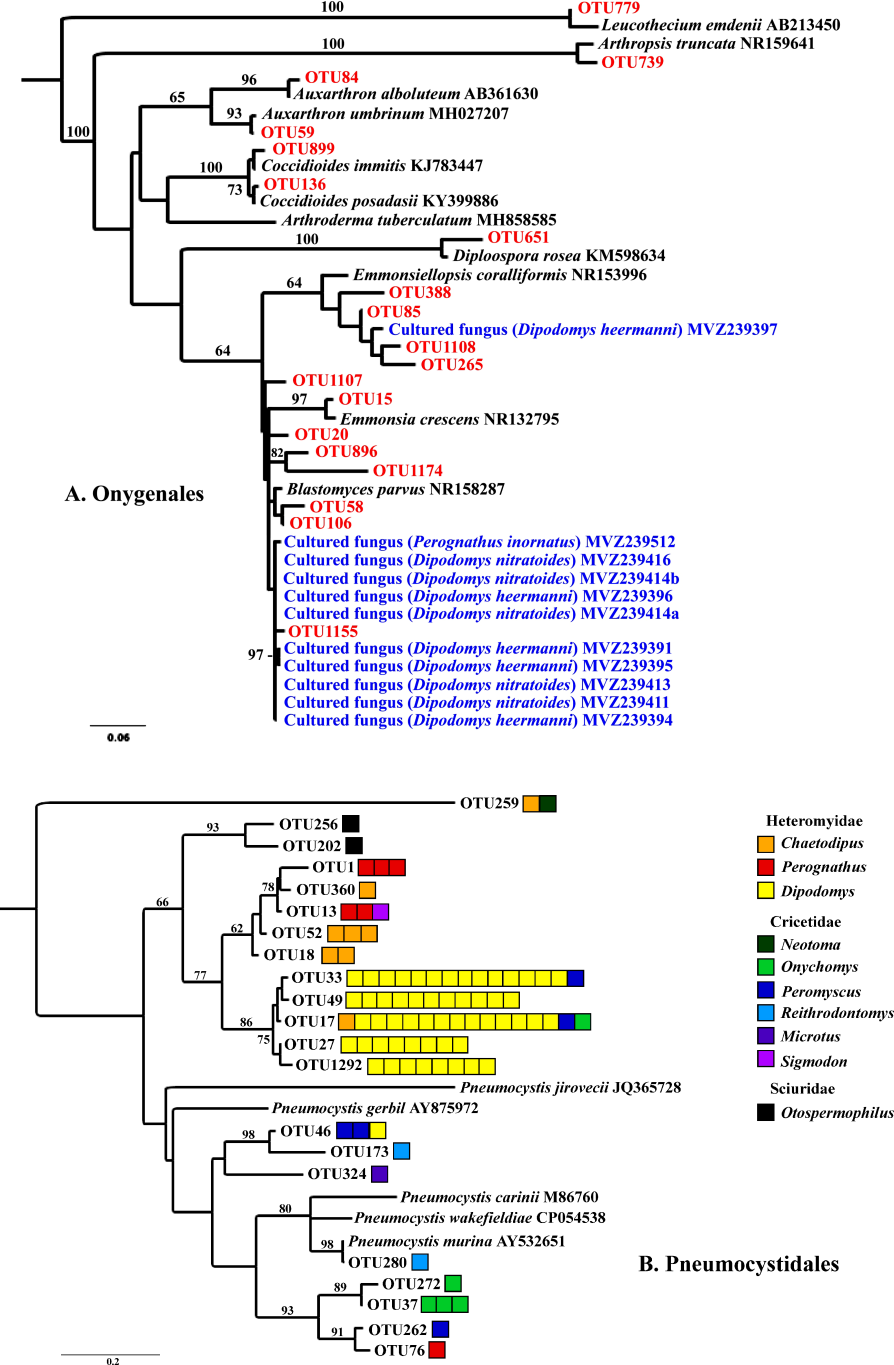
RaxML maximum likelihood phylogenies for Onygenales **(A)** and Pneumocystidales **(B)**. Both trees have a mid-point root and bootstrap values (1000 replicates) are shown for branches with greater than 60% support. **(A)** Onygenalean fungi obtained through culturing (blue) and Illumina ITS2 sequencing (red) of lung tissues. Host species for which lung fungal cultures were obtained are in parentheses. Sequences in black type were acquired from GenBank. **(B)** Pneumocystidales OTUs were obtained from Illumina ITS2 sequencing. Each box to the right of an OTU represents one sample with >1,000 sequence reads for a given OTU. Boxes are color coded by genus with warm colors (red, orange, yellow) within Heteromyidae, cool colors (green, blue, purple) within Cricetidae, and black for Sciuridae. Branches with species names and accession numbers represent sequences obtained from GenBank. These trees are presented to illustrate the diversity of fungi from these two groups that were obtained from lung samples, together with associations among cultured fungi, ITS sequences, and mammalian species. Based on ITS sequences alone we were not able to resolve deeper evolutionary relationships unambiguously.

We focused special attention on the frequency, abundance, and diversity within the orders Onygenales, Pneumocystidales, Malasseziales, and Saccharomycetales because of previous reports regarding the lung mycobiome. The Onygenales accounted for 19 OTUs and one or more of these OTUs were in 183 (92%) samples (**Table 1**; **Figure 2A**). The Malasseziales accounted for eight OTUs (all in *Malassezia*) and were present in 166 (83%) of the samples (**Table 1**). The Pneumocystidales were represented by 21 OTUs (all in *Pneumocystis*) and were in 165 (83%) of the samples (**Table 1**; **Figure 2B**). Members of the Saccharomycetales accounted for 13 OTUs and occurred in 75% of the samples.

We note that 13 OTUs had top hits to species of *Aspergillus*, and members of this genus occurred in 93% of the lung samples (**Table 1**). The most frequent and abundant of these, OTU3, had top BLAST GenBank hits to *A. fumigatus* (the primary cause of aspergillosis) and occurred in 67% of the lung samples (**Figure 1**). The difficulty in assessing the significance of sequences from *A. fumigatus* and other members of the Eurotiales in the context of the lung mycobiome is discussed below.

Many of the fungi inhabiting lung tissues have been designated as pathotrophs (**Supplementary Figure 1A**). When functional guild designations of animal pathogens and parasites were pooled, it was evident that most samples had a high abundance of known fungal animal symbionts, but there remains a large unclassified component (**Supplementary Figure 1B**).

Alpha diversity calculated by OTU richness, the Simpson index, and the Shannon index showed no differences among host family, state, or collection year (**Supplementary Figure 2**).

Rarefaction curves indicated substantial coverage of OTU diversity, but coverage varied among samples (**Supplementary Figure 3**).

**Figure 3:**
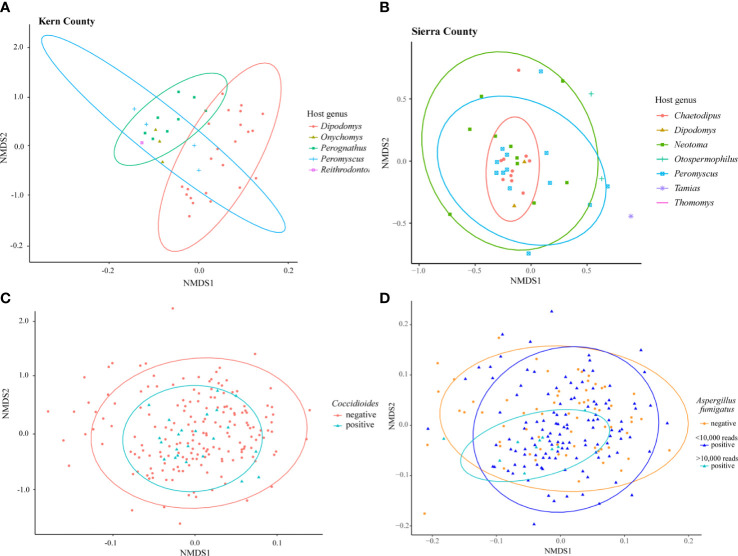
NMDS ordination of small-mammal samples from Kern County California only **(A)**, Sierra County New Mexico only **(B)**, and all 199 small-mammalian lung samples. **(A)** Samples collected within a 50 km radius in Kern County California suggest a difference in the lung mycobiome communities of species of *Dipodomys* and *Perognathus* (R^2^= 0.22905, p=0.001). **(B)** Samples within a 50 km radius in Sierra County New Mexico suggest a clustering of the lung mycobiome community within species of *Chaetodipus* but not differences from *Neotoma* or *Peromyscus* species (R^2^=0.07634, p=0.188). **(C)** Analysis of all 199 samples reveals no fungal community differences between those samples with *Coccidioides* and those without (R^2^=0.00539, p=0.351). **(D)** Analysis of all 199 samples demonstrated potential differences but also substantial overlap between those samples with OTU3 (top BLAST hits to strains of *Aspergillus fumigatus*) and those without (R^2^= 0.00883, p=0.005). A degree of clustering of samples with higher numbers of OTU3 reads (>10,000) was observed, however (indicated by the smaller turquoise oval). For the analysis shown, *A*. *fumigatus *sequence reads were removed to prevent the effect of abundant OTU3 on Bray-Curtis dissimilarity values. Results obtained when *A. fumigatus *reads were included in the analyses were in substance the same (R^2^= 0.01037, p=0.009, results not presented). Additional NMDS ordination results are presented for collection year, location by state, and host family and genus in **Supplementary Figure 3**.

Differences in fungal community composition among samples were evaluated using nonmetric multidimensional scaling (NMDS) ordinations with the Bray-Curtis dissimilarity metric. There were no clear trends (p>0.05) in the fungal communities across time (collection year or month), location (state), or host (genus or family) although limited sample size may have dampened visible patterns (**Supplementary Figure 4**). Subsets of the data based on well sampled host families were analyzed to explore small spatial scale patterns. PERMANOVA was used to test for statistical differences between groups. In Kern County, California, 40 samples collected from four localities within a 50 km radius showed a clear separation existed in fungal community composition between the host genera of *Dipodomys* and *Perognathus* (**Figure 3A**, R^2^=0.22905, p=0.001). A similar pattern was not observed for Sierra County, New Mexico; while there was clustering of *Chaetodipus* samples, *Dipodomys* and *Perognathus* sampling was not sufficient to make direct comparisons with Kern County (**Figure 3B**, R^2^=0.07634, p=0.188). The lung communities in samples with *Coccidioides* present were not significantly different from those without *Coccidioides* sequences (**Figure 3C**, R^2^=0.00539, p=0.351). In NMDS analyses, lung samples with OTU3 (top BLAST hits to strains of *Aspergillus fumigatus*) substantially overlapped those without this OTU even though a PERMANOVA analysis suggested differences between the two sample classes (R^2^=0.00883, p=0.005). When only samples with >10,000 OTU3 reads were considered there was evidence of clustering for these samples (**Figure 3D**).

A Mantel test revealed significant positive correlation between geographic distance and fungal community dissimilarity (r=0.1132, p=0.001). Positive spatial autocorrelation was seen at two distance classes, less than 44.8 km and between 135 km and 224 km indicating that samples collected closer together tend to have more similar fungal communities (**Supplementary Figure 5A**). Other distance classes had no positive or negative autocorrelation. Although lung fungal communities in general exhibited low Bray-Curtis similarity in pairwise comparisons, distance-decay analysis indicated significant decrease in similarity with distance (p<0.001; **Supplementary Figure 5**).

### Coccidioides, Blastomyces parvus, and other Onygenales

**Figure 4:**
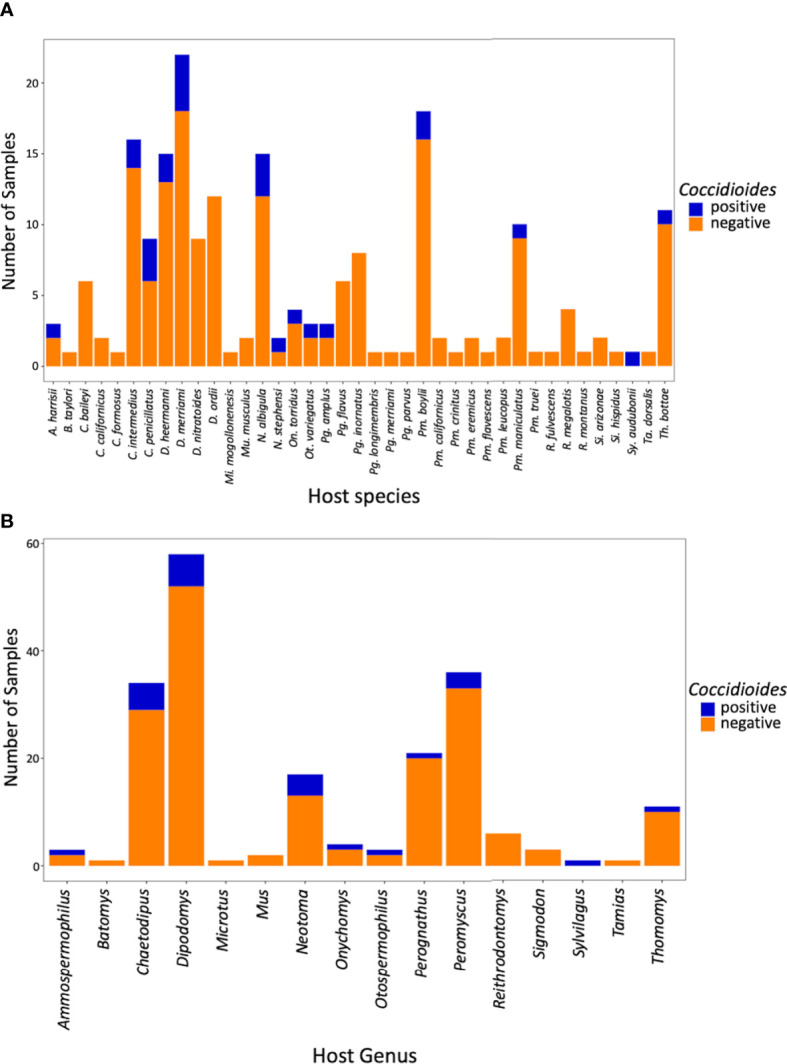
Bar plot of *Coccidioides* positive and negative samples by species **(A)** and by genus **(B)**.

**Figure 5:**
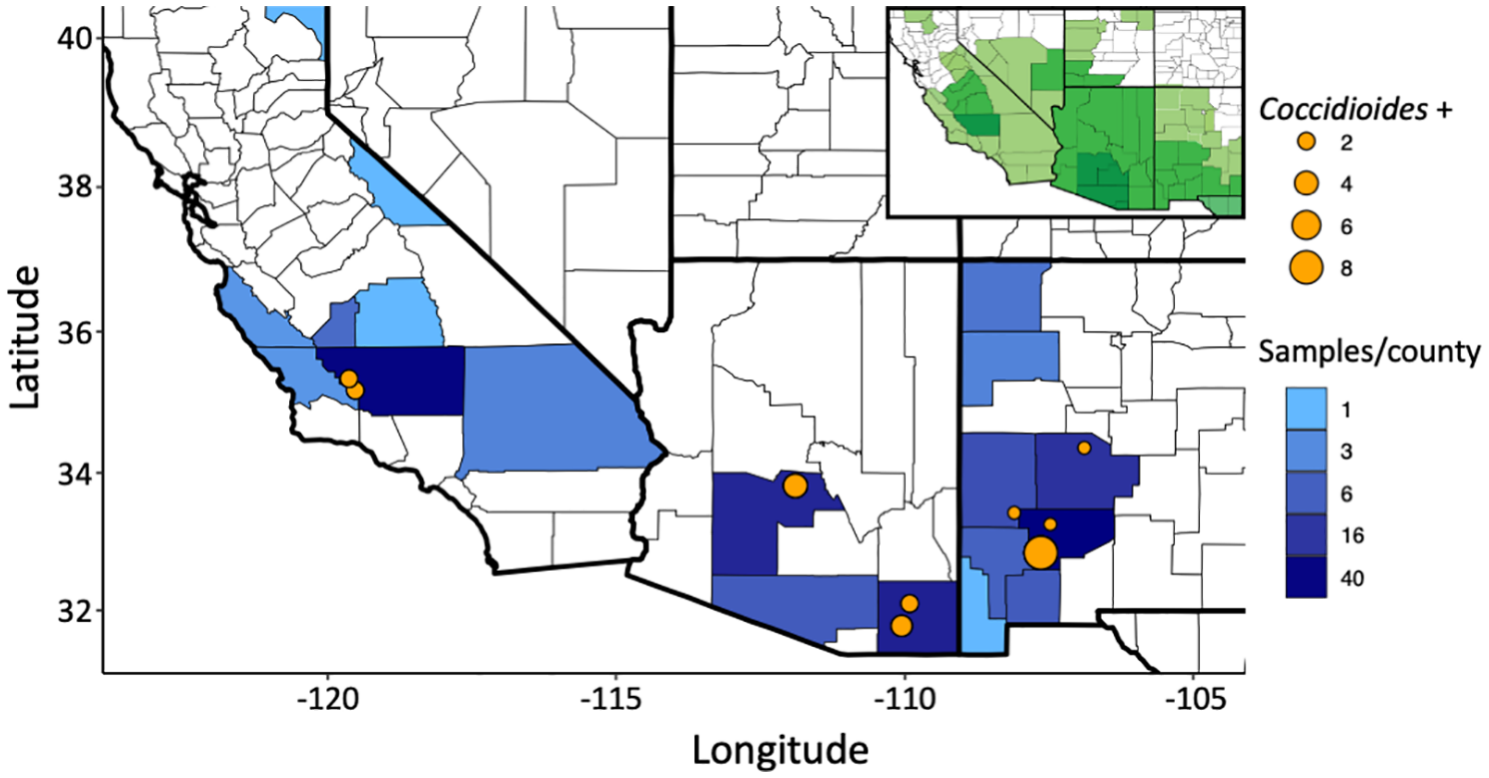
Sampling of small-mammalian lungs across the southwestern U.S. detect *Coccidioides* in wild animals. The number of samples sequenced per county is depicted in blue. *Coccidioides* positive trapping locations are shown with an orange dot with increasing size based on the number of positive samples in that specific location. GPS locations less than 1 km apart were merged. The insert indicates the endemic range for the genus *Coccidioides*, with darker shades of green indicating higher levels of incidence, as recognized by the U.S. Centers for Disease Control and Prevention (see Gorris et al., 2019).

Of the 199 small mammalian lungs for which we obtained Illumina ITS2 sequences, 24 (12%) produced *Coccidioides* sequence reads. These *Coccidioides*-positive samples fell within 14 mammalian species (*Ammospermophilus harrisii*, *Chaetodipus intermedius*, *Cheatodipus penicillatus*, *Dipodomys heermanni*, *Dipodomys merriami*, *Neotoma albigula*, *Neotoma stephensi*, *Onychomys torridus*, *Otospermophilus variegatus*, *Perognathus ampulus*, *Peromyscus boylii*, *Peromyscus maniculatus*, *Sylvilagus audubonii*, and *Thomomys bottae*) representing 10 genera and 5 families (Cricetidae, Heteromyidae, Sciuridae, Geomyidae, and Leporidae) (**Figure 4**; **Supplementary Table 2**). Positive *Coccidioides* samples were found in California (Kern County), Arizona (Cochise and Maricopa Counties) and New Mexico (Catron, Sierra, and Socorro Counties) (**Figure 5**; **Supplementary Table 2**). *Coccidioides* positive rates were highest in Maricopa County, Arizona (27%) and Sierra County, New Mexico (20%). UPARSE analysis produced two *Coccidioides* OTUs (OTU136 and OTU899) that were 97.38% similar. OTU136 shared a closer sequence similarity to *C. posadasii* and OTU899 shared a closer sequence similarity to *C. immitis* (**Figure 2A**).

Seven OTUs exhibited best hits to *Blastomyces parvus* in GenBank BLAST searches (**Figure 2A**). One or more of these OTUs occurred in 85% of the lungs sampled. *Blastomyces* OTU20 occurred in more than 80% of the samples and was the third most frequent OTU in the community data (**Figure 1**). Two other variants with top BLAST hits to *B. parvus*, OTU106 and OTU20, each occurred in more than half of the lungs sampled (**Figure 1**). Co-occurrence analyses revealed no significant correlation between the occurrence of members of the *B. parvus* group and species of *Coccidioides* (rho= -0.0257, p=0.6351, **Supplementary Table 3**).

### Cultured members of the Onygenales

Although for most lung samples we did not have sufficient tissue for culture plating, we plated tissues from the lungs of 25 animals that were collected in 2019 during this study. This was done in part to test whether we could recover *B. parvus* and other members of the Onygenales, after preliminary sequencing efforts suggested the frequent presence of these fungi. Isolates whose top BLAST hits were to *B. parvus* were the most frequent among isolates obtained by direct culturing of lung tissues (11 of 29 isolates, **Figure 2A**; **Supplementary Table 4**).

Several OTUs had best BLAST GenBank hits to species in other Onygenalean genera, including *Emmonsia*, *Emonsiellopsis*, *Auxarthron*, *Diploospora*, *Arthropsis*, and *Leucothecium* (**Figure 2A**). Among these latter genera, only a species of *Emmonsiellopsis* was obtained in culture from mammal lungs (**Figure 2A**).

### Pneumocystidales diversity

The 21 OTUs assigned to *Pneumocystis* were present in rodents of the families Heteromyidae, Cricetidae, and Sciuridae with patterns suggesting substantial but not complete host specificity (**Figure 2B**). Phylogenetic relationships among fungal OTUs from the Heteromyidae were substantially congruent with established relationships for the three mammalian genera, namely showing a closer relationship between *Chaetodipus* and *Perognathus* relative to *Dipodomys* (Alexander and Riddle, 2005). *Pneumocystis* sequences were especially prevalent in species of *Dipodomys* (32 of 57 samples, 56%), and were less frequently observed in species of *Peromyscus* (4 of 36 samples, 11%). Of the 165 samples containing *Pneumocystis*, 23 (14%) had multiple OTUs with >1000 reads each.

### Additional fungi cultured from lung tissues

A total of 29 fungal isolates were obtained from five species of rodents from Kern County, California (**Supplementary Table 4**). In addition to isolates of the Onygenales referenced above, isolates included *Aspergillus fumigatus*, *Aspergillus* sp., several isolates of *Penicillium* sp., two members of the Chaetomiaceae, and two members of the Mucorales. As mentioned above, sequences from *A. fumigatus* were both frequent and abundant in the ITS2 Illumina data.

## Discussion

Our Illumina sequencing results produced an average of just over 40 fungal OTUs per lung sample and more than 700 OTUs across the 199 samples examined (**Supplementary Data Sheet 2**). The following discussion assumes minimal contamination of samples from natural and laboratory environments. As described in Materials and Methods, all PCR-based experiments included negative controls. In addition, while the lung samples employed were collected over a period of twenty-five years by multiple scientists, they were obtained using sterile protocols designed to minimize contamination (Yates, 1996; Yates et al., 1996; Galbreath et al., 2019). The problem of potential environmental and laboratory contamination has received special interest in the context of species of *Malassezia*, once thought to be restricted to skin but now recognized as common in diverse microbiome and environmental samples unrelated to contamination (Spatz and Richard, 2020). While the potential for contamination by species of *Malassezia* and other environmental fungi provide a caution for interpreting results of microbiome studies in general, we note that the fungi we have focused on here, especially the Onygenales and Pneumocystidales, are not common in environmental samples.

We recognize four groups of fungi that occur in lung tissues: 1) those present because of incidental inhalation of spores but not truly colonizing lung surfaces or tissues; 2) fungi that are capable of colonizing lung tissue as commensals or pathogens transiently or long-term but are not specifically adapted to the lung; 3) fungi that are common members of the mammalian mycobiome that can exist in lung tissues, but which can also be associated with other tissues such as skin or gut, and 4) fungi adapted to the lung, either obligately (*Pneumocystis* species) or facultatively (*Coccidioides* species, *Aspergillus fumigatus*). These categories will likely be fluid depending on the fungi and mammals in question, for example because of potential differential susceptibility of mammalian species to pathogenicity.

The two most difficult mycobiome groups to distinguish between are those representing incidental inhalation (group 1) and transient colonization (group 2). Fungi present from incidental inhalation might be expected to occur in fewer samples and/or exhibit low read numbers, and their sequences would likely be common in environmental surveys. Such fungi could include sporulating members of the Sordariales, Helotiales, Pezizales, Dothidiales, Eurotiales, Hypocreales, Agaricales, and Pleosporales (Klich, 2002; Fröhlich-Nowoisky et al., 2009; Porras-Alfaro et al., 2011; Hamm et al., 2020a). A study by Kramer et al. of the airway mycobiome of cystic fibrosis patients found that sequences from many fungal species had high fluctuations both among different patients and over time when patients were sampled multiple times, suggesting the pulmonary mycobiome was dominated by species present temporarily because of inhalation of environmental spores (Kramer et al., 2015). Similarly, Rubio-Portillo et al. reported substantial variation among the mycobiomes of human bronchoalveolar lavage samples from different individuals, while in contrast they observed correlations between fungal OTUs from lavage samples and air samples from the home environments of test subjects (Rubio-Portillo et al., 2020). This further supports the potential relevance to lung mycobiome studies of incidental inhalation of spores from the local environment.

The importance of transient colonization (group 2), though difficult to separate from simple incidental inhalation in any specific instance, can be inferred from the fact that common environmental fungi not generally thought of as human pathogens occasionally cause disease, especially in immunocompromised individuals. Such fungi include species of *Fusarium*, *Penicillium*, *Alternaria*, and many others including members of the Mucoromycota (Skiada et al., 2017). And although the immune system of healthy individuals efficiently works against such infections, the triggering of defensive responses may in fact depend on transient colonization (Hernández-Chávez et al., 2017; Gago et al., 2019). With respect to our results, the strong correlation between read abundance and frequency (**Figure 1**) might support the argument that certain fungi, for example members of the Eurotiales not generally recognized as pathogens, are capable of colonization of healthy lung tissue beyond simply being present passively because of inhalation. This could include those species of *Aspergillus* that only rarely cause serious infections (Tsang et al., 2020).

We also acknowledge that *A. fumigatus*, sequences of which were common in our dataset (based on BLAST hits), is special in that it causes the most frequently diagnosed lung mycosis and is at the same time a common environmental saprotroph. *Aspergillus fumigatus* has multiple adaptations that permit colonization of lung tissues, including small spore size, thermotolerance, and molecular mechanisms that allow avoidance of immune responses (Robert and Casadevall, 2009; Casadevall, 2012; Kwon-Chung and Sugui, 2013; Raffa and Keller, 2019); and in that sense it might fit into either group 4 or group 2. Currently, it is difficult to know for certain whether the apparent frequent occurrence of *A. fumigatus* in the lungs of small mammals reflects the large number of spores in the environment or colonization of lung tissues. However, the fact that in NMDS analyses samples with high read counts for OTU3 (presumed *A. fumigatus*) formed a cluster relative to samples with low and no OTU3 reads (**Figure 3D**) may provide support for colonization in such samples.

Taken as a whole, our results have similarities and differences with respect to results obtained for the lung mycobiome of humans. Fungi commonly detected in healthy human lungs have included *Candida* (Saccharomycetales) *Malassezia* (Malasseziales), *Cladosporium* (Cladosporiales), *Penicillium* and *Aspergillus* (Eurotiales), and *Pneumocystis* (Tipton et al., 2017). Similarly, OTUs representing the Malasseziales and Saccharomycetales (including *Candida*), commonly associated with the human mycobiome (group 3), were well represented in our data (83% and 49% of the samples, respectively; **Figure 1**; **Table 1**). The relatively high diversity of Malasseziales fungi (eight OTUs) in our samples is similar to results reported previously for both healthy and diseased human lungs (van Woerden et al., 2013; Willger et al., 2014; Fraczek et al., 2018).

In our results, many OTUs were both abundant in sequence reads and frequency across samples despite being from different small mammals and various locations (**Figure 1**). This contrasts with results reported for humans that indicated high inter-individual variability for many OTUs (Kramer et al., 2015; Rubio-Portillo et al., 2020). In addition, sequences from members of the Onygenales were frequent in our samples but have been rare or non-existent in human samples. One potential contributing factor to these differences between our study and human studies is that we sampled lung tissues as opposed to bronchoalveolar lavage or sputum, and as a result our samples have the potential to detect fungi more deeply in tissues than would be obtained with lavage or sputum sampling. Another potential difference could reflect the fact that small, fossorial mammals spend substantial amounts of time in close contact with the soil environment, which could increase exposure to fungi in the soil environment, thereby reflecting more intense sampling of fungi in soil.

The most compelling results from our study relate to the Pneumocystidales and Onygenales, offering new insights into the biology of these organisms. Species of *Pneumocystis* have long been reported to be common obligate lung fungi with substantial host specificity, and most species are yet to be formally described (Mazars et al., 1997; Demanche et al., 2001; Laakkonen et al., 2001; Akbar et al., 2012). Members of this group possess genomic and physiological adaptations to be obligate biotrophs of mammals (Ma et al., 2016), and they clearly belong to the group of fungi adapted to the lung (group 4). Surveys for species of *Pneumocystis* in the lungs of non-human primates and bats using specific *Pneumocystis*-directed PCR produced positive results in 30-40% of the lung tissues examined (Demanche et al., 2001; Akbar et al., 2012), although one study reported a high incidence in shrews (80% of 83 samples; Danesi et al., 2020). Our Illumina approach resulted in 83% of the samples (165 of 199) containing sequences from members of this group. This percentage is almost certainly an underestimate, given that we examined only small amounts of lung tissue from each animal. Our results suggest that in some instances a single lung can harbor multiple *Pneumocystis* lineages, similar to results reported for *P. carinii* and *P. wakefieldiae* (Cushion et al., 2004; Latinne et al., 2021). For example, several different individuals of *Dipodomys heermanni* had more than 1000 reads assigned to both OTUs 27 and 1292. Moreover, in some instances our data suggest the possibility of lineage spillover across rodent families (**Figure 2B**). These are congruent with the findings of Latinne et al. (2021) obtained for Southeast Asian rodents indicating that certain *Pneumocystis* lineages may not be restricted to a single host. Nevertheless, host specificity of *Pneumocystis* species and their animal hosts is apparent for multiple genera of Heteromyidae especially *Dipodomys* (**Figure 2B**). The evolution of *Pneumocystis* host niche is complex and not well understood (Babb-Biernacki et al., 2020). Our study suggests that high-throughput non-targeted sequencing can be valuable to gain information toward understanding *Pneumocystis* diversity and host specificity.

Our results support the hypothesis that certain Onygenalean fungi, including species of *Coccidioides* and members of the *Blastomyces parvus* group, are adapted to infect and persist in lung tissues. Taylor and Barker have argued that species of *Coccidioides* are adapted to be endozoan inhabitants of small-mammal lungs rather than soil fungi with the ability to cause opportunistic infections (Taylor and Barker, 2019). This argument is based in part on genomic studies of *Coccidioides* species and other members of the Onygenales that showed a reduction of genes involved in plant decomposition and an increase in genes involved in the degradation of animal proteins (Sharpton et al., 2009). Moreover, sequences from species of *Coccidioides* and *Blastomyces* are rare in environmental surveys including studies that sample sites where species of *Coccidioides* are endemic (Porras-Alfaro et al., 2011; Bates et al., 2012), and the detection of *Coccidioides* species in soils is sporadic even with robust methods (Greene et al., 2000; Barker et al., 2012). Further, OTUs from the Onygenales typically do not have close relatives among sequences from environmental samples (uncultured fungi) in GenBank. This paucity of representation in environmental surveys contrasts with the diversity (19 OTUs) and prevalence (92%) of Onygenalean OTUs in the lung tissues examined here. An illustration of this disparity is found in a comparison of BLAST hits at GenBank using the eight most frequent Eurotiales OTUs in our dataset with those obtained with the eight most frequent Onygenales OTUs (**Supplementary Table 5**). The Eurotiales had equally good hits to GenBank entries from environmental samples and sequences obtained from known organisms. In contrast, the Onygenales sequences produced close matches to sequences from known organisms but not to environmental sequences.

Although coccidioidomycosis has been reported in diverse animal species (del Rocío Reyes-Montes et al., 2016), there are only two studies reporting the direct isolation of *Coccidioides* species from wild small mammals. One reported cultured *Coccidioides* from pocket mice (*Chaetodipus intermedius, C. penicillatus*, and *C. baileyi*, reported as *Perognathus* in the literature but later reassigned to *Chaetodipus*; Hafner and Hafner, 1983), the southern grasshopper mouse (*Onychomys torridus*), and a Merriam’s kangaroo rat (*Dipodomys merriami*) trapped in Arizona (Emmons and Ashburn, 1942). In Baja Mexico, a serology assay detected coccidioidal antibodies in a deer mouse (*Peromyscus maniculatus*) and desert woodrat (*Neotoma lepida*; Catalán-Dibene et al., 2014). Our ITS2 data represent the first high-throughput sequencing approach to attempt detection of *Coccidioides* species in wild small-mammal lungs. Here, we report 14 species of small mammals with sequences from *Coccidioides*, nine of which (*Ammospermophilus harrisii*, *Dipodomys heermanni*, *Neotoma albigula*, *Neotoma stephensi*, *Otospermophilus variegatus*, *Perognathus ampulus*, *Peromyscus boylii*, *Sylvilagus audubonii*, and *Thomomys bottae*) are newly documented host species (**Figure 4A**).

Early research from Emmons and colleagues suggested a higher correlation with heteromyid rodents and *Coccidioides* in the environment (Emmons and Ashburn, 1942; Emmons, 1943). Within the Heteromyidae rodents in our study, the genus *Chaetodipus* had a 15% positivity rate and *Dipodomys* had a 10% positivity rate, but they did not greatly differ from our overall average *Coccidioides* detection frequency (12%) (**Figure 4B**). In terms of future studies, *Neotoma* (woodrats) may be of particular interest with a *Coccidioides* positive rate of 24%, two-fold higher than our overall average (**Figure 4B**). An overlap analysis between the distribution models of rodents and species of *Coccidioides* has suggested that *Neotoma lepida* is a predominant co-occurring species (Ocampo-Chavira et al., 2020).

We were limited by sample size in terms of the Leporidae, Sciuridae, and Muridae. Notwithstanding, one *Sylvilagus audubonii* (desert cottontail) sample was positive for *Coccidioides* (**Figure 4A**). *Sylvilagus audubonii* DNA was found in association with *Coccidioides* positive burrows in Arizona (Kollath et al., 2019). Despite having only five samples of sciurids (squirrels), we detected *Coccidioides* in two genera (*Ammospermophilus* and *Otospermophilus*) corresponding to 40% of the samples. Only two murid samples were obtained, and neither were positive for *Coccidioides*.

With the expected expansion of coccidioidomycosis endemic regions in the United States (Lauer, 2017; Gorris et al., 2019), efforts have increased to model the factors affecting the distribution of the disease and predict its expansion under climate change scenarios. These efforts have, however, focused on environmental factors (climate, soil, elevation, and land cover variables) to predict ecological niches for *C. immitis* and *C. posadasii* (Baptista-Rosas et al., 2007; Gorris et al., 2018; Weaver et al., 2020; Dobos et al., 2021). Our study supports the prediction that small mammals play important roles as host reservoirs of *Coccidioides* species (del Rocío Reyes-Montes et al., 2016; Taylor and Barker, 2019), suggesting that the ecology, distribution, and taxonomy of small mammals should be incorporated into *Coccidioides* modeling efforts.

The presence of *Coccidioides* sequences does not appear to disrupt the lung mycobiome (**Figure 3C**). Studies with chytridiomycosis in frogs (Jani and Briggs, 2014) and snake fungal disease (Allender et al., 2018) suggest that these infections cause a disruption of the microbiome and a decrease in both fungal and bacterial diversity with fungal pathogen introduction. In sea star wasting disease, changes in microbial community composition occurred during disease progression, with decreasing species richness only in the late stages of the disease (Lloyd and Pespeni, 2018). The presence of *Coccidioides* without mycobiome disruption might imply a primarily commensal relationship with its host until a change in host conditions favoring a pathogenic state.

Emmons suggested a correlation between the presence of *Blastomyces parvus* (previously *Haplosporangium parvum*) and *Coccidioides* stating, “The isolation of *H. parvum* from rodents of a given area is taken to indicate that *C. immitis* is probably also present in that area” (Emmons, 1943). Our ITS2 sequencing revealed *Blastomyces* sequences in 85% of the samples suggesting that it is more common in small-mammal lungs than are species of *Coccidioides* (12%). When abundances were rarified, *Blastomyces* was present without *Coccidioides* in 77 samples, but only co-occurred with *Coccidioides* in 6 samples. A Spearman correlation and a permutation test found no statistical correlation between the two Onygenalean genera (**Supplementary Table 3**). Even without correlation, it is notable that *Blastomyces parvus* is in high frequency in small-mammal lungs despite its ability to cause adiaspiromycosis (Emmons and Ashburn, 1942). *Emmonsia crescens* (OTU15), detected in 16% of the samples, is an additional etiological agent of adiaspiromycosis (**Table 1**, **Figure 2A**) (Emmons and Jellison, 1960). Adiaspiromycosis often presents as a pulmonary infection common to fossorial mammals like rodents but is much rarer in humans (Anstead et al., 2012).

Lung samples employed in this study were chosen to reflect the geographic range of human coccidioidomycosis from low to high incidence to determine if we could detect sequences from species of *Coccidioides*. One of the most revealing aspects of our results, however, had to do with the high frequency of sequences from *B. parvus* and other members of the Onygenales among lung samples (92%). In addition, *B. parvus* was the most common fungal species cultured from lung tissue. We argue that the endozoan, small mammal reservoir hypothesis (Taylor and Barker, 2019) should be expanded beyond species of *Coccidioides* to encompass multiple Ajellomycetaceae (Onygenales) fungi including *Blastomyces*, *Emergomyces*, *Emmonsia*, and *Emmonsiellopsis*, an argument with significant clinical relevance. The phylogenetic diversity within this group is becoming ever more important to the medical field as over the last four decades there has been an increase in reports of novel *Emmonsia*-like human pathogens (Schwartz et al., 2015). Our study and others (Danesi et al., 2020) demonstrate the commonality of these *Emmonsia*-like fungi globally in wild animals. Host jumps have allowed and will likely continue to allow these fungi to evolve and diversify followed by radiation, specialization, and speciation which in some cases could increase their virulence (Thines, 2019).

This study was made possible by the frozen tissue collections of the UNM Museum of Southwestern Biology and the UCB Museum of Vertebrate Zoology. While acknowledging the value of the availability of diverse, frozen tissue collections, we also note that molecular barcoding of specimens to confirm host identifications is best practice (Dunnum et al., 2017). About 11% of the samples in this study showed incongruence between host identification based on cyt b sequences and that reported in museum databases. Nine of these frozen samples can be attributed to tissues that were apparently mislabeled at the time of collection, while 13 samples represent misidentification of closely related cryptic species for which morphology alone is inconclusive. Misidentification based on morphological features alone has been reported to be as high as 10% in some pathogen studies (Müller et al., 2013); however, without the ability to both test the host identity with a molecular approach and return to a physical voucher, these kinds of incongruencies would have been impossible to rectify. Directly linking genetic data to a physical specimen should become a necessary component of infectious disease studies (Thompson et al., 2021).

## Materials and methods

### Tissue acquisition

Ultra-frozen lung tissues were obtained from the University of New Mexico Museum of Southwestern Biology (MSB) and the University of California Berkeley Museum of Vertebrate Zoology (MVZ) by formal request. Sampling ranged from 1994 to 2019. Archival tissues loaned to this project included 39 species within five families (Heteromyidae, Cricetidae, Muridae, Sciuridae, and Geomyidae) of rodents and one rabbit species in Leporidae (**Supplementary Table 1**). Field sampling procedures followed established, sterile protocols developed to avoid cross-contamination and maximize the utility of these collections (Yates, 1996; Yates et al., 1996; Galbreath et al., 2019). Typically, specimens were preserved under a series of three-year protocols approved through the Institutional Animal Care and Use Committees (IACUC). The current protocol (Animal Welfare Assurance # D16-00565; A4023-01) is enforced under the United States Department of Agriculture Registration # 85-R-0002. Upon sacrifice of the mammal specimen following approved guidelines for animal care and use (Sikes et al., 2016), lung samples were collected into cryovials, immediately flash frozen in liquid nitrogen, and then archived and data-based at the ultrafrozen facilities at MSB and MVZ. Samples were obtained from 45 sampling localities from 19 counties in California, Arizona, and New Mexico within the known distribution of coccidioidomycosis (**Figure 5**).

### DNA purification from lung tissues

Approximately 0.025 g of lung tissue was lyophilized for 24 hours followed by DNA extraction using the following CTAB procedure. Tissue was resuspended and ground in 500 µL cetyltrimethylammonium bromide (CTAB) lysis buffer (2% CTAB, 1.4 M NaCl, 20 mM EDTA, 100 mM Tris–HCl) plus *β*-mercaptoethanol (final concentration 0.2%) and 10 µL protease K (10 mg/mL) followed by an hour at 65°C. An isoamyl alcohol/chloroform extraction was performed by adding 500µL isoamyl alcohol-chloroform (1:24) followed by 20 minutes of gentle shaking and then centrifugation at 16,000g for 5 minutes. The upper aqueous phase was transferred to a fresh tube. DNA was precipitated by addition of 15 µL 3M sodium acetate (pH 5.2) and 500µL ice-cold isopropanol. Samples were inverted and incubated at -20°C for 10 minutes. After centrifugation, the pellet was washed twice, first with 500µL ice-cold 70% ethanol and then with 500µL ice-cold 100% ethanol. Ethanol was discarded and the pellet dried prior to resuspension in 50µL sterile H_2_O. DNA was further purified with Agencourt AMPure beads (Agencourt Bioscience Corporation, Beverly, MA, USA) following instructions from the manufacturer.

### Host identifications

Host species were tentatively identified in the field using morphological parameters including but not limited to total length, tail length, hind foot length, ear length, reproductive data, total weight, and coat coloration. Geographic locality and field guides were additionally used for verification. To check species designations, we partially sequenced the mitochondrial cyt b region. Purified DNA extracted from lung tissue was used to amplify the cyt b region with primers targeting specific host groups. Cricetids (*Neotoma*, *Peromyscus*, *Onychomys*, *Reithrodontomys*, *Baiomys*, and *Sigmodon*), and murids (*Mus*) were amplified with the MSB05 and MSB14 modified primers as described in (Hope et al., 2010). Heteromyids (*Chaetodipus*, *Dipodomys*, and *Perognathus*), geomyids (*Thomomys*), and sciurids (*Ammospermophilus*, *Tamias*, and *Otospermophilus*) were amplified with the MVZ05 and MVZ14 primer pair (Smith and Patton, 1993). If the initial amplification was unsuccessful, we employed primer pair MVZ05 and MVZ04 which amplifies a shorter 426 bp segment (Smith et al., 1992). Polymerase chain reaction (PCR) conditions were as follows: initial denaturation at 95°C for 10 min, followed by 34 cycles of 95°C for 15 sec, annealing at 52°C for 30 sec, and extension at 72°C for 1 min, with a final extension at 72°C for 5 min, and holding at 4°C. PCR was followed by gel electrophoresis confirmation. Products were purified with ExoSAP-IT (Affymetrix, Santa Clara, CA) according to the manufacturer’s recommendations followed by Sanger sequencing using BigDye Terminator v3.1(Applied Biosystems, Foster City, CA) employing the forward and reverse primers. Identification was performed using BLAST searches. Cyt b sequences were deposited in GenBank (Sayers et al., 2021) under accession numbers OK134972 to OK135142 (**Supplementary Table 1**). For 43 samples that could not be verified with molecular methods, we utilized the archived host identification (https://arctos.database.museum).

### Illumina library preparation and sequencing

The preparation of amplicon libraries was preceded by a PCR amplification using primers ITS1-F and ITS4 (White et al., 1990; Gardes and Bruns, 1993) to amplify the fungal nuclear ribosomal ITS region from whole-lung DNA. These reactions were performed in 15 µL reactions with 7.5 µL Premix ExTaq polymerase (Takara, Mountain View, CA), 1 µL of each (5 µM) primer, 2 µL of 1 mg/mL bovine serum albumin, 2.5 µL of sterile water and 1 µL of template DNA. PCR products were checked by gel electrophoresis. DNA concentration of successful amplifications were measured with a Qubit dsDNA HS kit (Life Technologies Inc., Gaithersburg, MD) and serial dilutions were performed to dilute inhibitors. A second PCR was preformed to contain a 29 (forward) or 25 (reverse) base linker, a 12 base barcode, a 29 (forward) or 34 (reverse) base pad, a 0-8 base heterogeneity spacer, and the fungal ITS2 5.8S-FUN and ITS4-FUN primers (Taylor et al., 2016), following the procedure in (Gao et al., 2019). Negative controls were included with all PCR procedures to ensure that amplified fragments did not result from laboratory contamination. PCR products were purified using AMPure magnetic beads (Beckman Coulter Inc., Brea, CA) following the manufacturer’s instructions and pooled to create DNA libraries following the Illumina MiSeq DNA library preparation protocol for paired-end reads. Libraries were quality checked for concentration and amplicon size using the Agilent 2100 Bioanalyzer (Agilent Technologies, Santa Clara, CA) at the Vincent J. Coates Genomics Sequencing Laboratory (GSL, University of California, Berkeley, CA). Pyrosequencing was performed on the Illumina MiSeq PE300 sequencing platform at the GSL. All raw reads were deposited in the NCBI Short Read Archive (SRA) under BioProject ID PRJNA769405.

### Sequence processing

Illumina sequences were processed initially with USEARCH v11 (Edgar, 2013). Sequences less than 250 bases in length were removed, as were adapter and primer sequences, and presumed chimeras. Sequences were clustered into OTUs at 99% similarity with UPARSE implemented in USEARCH (Edgar, 2010). Reports of nearly identical outcomes for community analyses utilizing OTUs and ASVs (Glassman and Martiny, 2018; Joos et al., 2020) support our choice of OTUs to avoid overestimating the number of fungal lineages due to intragenomic and intraspecific variation among ribosomal DNA repeats in the ITS2 region (Schoch et al., 2012; Estensmo et al., 2021). Representative OTU sequences were assigned taxonomy initially using SINTAX (Edgar, 2016) and the UNITE database v8.2 (Nilsson et al., 2019), supplemented with GenBank BLAST searches for taxonomic confirmation. The results presented in **Figures 1**, **2** and **Table 1** reflect taxonomy confirmed with searches at GenBank. Fungal sequences from representative OTUs were deposited in GenBank under the accession numbers OK078030 to OK078530 (**Supplementary Data Sheet 2** links OTUs and accession numbers). Code can be found at https://github.com/p-salazarhamm/mammal_lung_mycobiome.

### Community and spatial analyses

We visualized differences in lung mycobiome composition among small mammals across time, space, and host using two-dimensional NMDS ordinations, a method commonly employed in microbial ecology to evaluate dissimilarities among communities (Legendre and Legendre, 2012). We transformed the data to relative abundance by dividing OTU read counts by the total of all reads within a sample. Ordinations were then performed on the relative abundance transformed fungal community data using the Bray-Curtis dissimilarity metric in the phyloseq package v1.30.0 (McMurdie and Holmes, 2013). We created subsets of the data and generated ordinations for intensely sampled counties to assess differences in fungal community by host on small spatial scales.

Spatial structure was examined by using a Mantel test in the vegan package v2.5-7 (Oksanen et al., 2019) in R v3.5.1 (R Core Team, 2018) to investigate a correlation between geographic distance and sample similarity. To assess the spatial distances at which communities were more or less similar than expected due to chance, we used a Mantel correlogram also in the vegan package. Furthermore, we plotted distance decay of similarity by plotting spatial distance between each pair of samples against the Bray-Curtis similarity between those samples.

### Functional analyses

We used FUNGuild v1.1 (Nguyen et al., 2016) to assign trophic modes and guilds (functional groups) to fungal OTUs. Animal-fungal symbionts per sample were pooled by determining the percent of reads in each sample that belonged to OTUs whose guild assignments included animal pathogen and animal parasite.

### Alpha diversity

OTU richness, the Simpson index, and the Shannon index were calculated to assess differences in alpha diversity of samples by location (state), host family, and collection date. To account for differing sequencing depths among samples, expected richness at 1000 reads was calculated using the *rrarefy* function in the vegan package (Oksanen et al., 2019). We used 1000 reads as a cutoff to retain reads from species of *Coccidioide*s, which were in low abundance. We additionally computed the rarefaction curve to determine OTU richness at varying sequencing depths.

### Co-occurrence analysis

Abundance data was rarified at three different read depths using the *rrarefy.perm()* function for computing 1000 rarefactions using EcolUtils R package (https://github.com/GuillemSalazar/EcolUtils). The patterns held consistent at the various depths (**Supplementary Table 3**). Seven OTUs (OTU20, OTU58, OTU1155, OTU1107, OTU106, OTU1174, and OTU896) identified as *Blastomyces* were grouped together to determine their co-occurrence with the two OTUs of *Coccidioides* (OTU136 and OTU899). The observed Spearman correlation was calculated. To test for significance, a permutation test was run for 10000 random draws and the observed rho statistic was compared to the corresponding null distribution.

### Phylogenetic analyses

Illumina sequences representative of OTUs from the Onygenales and Pneumocystidales along with representative sequences from GenBank were subjected to phylogenetic analyses. Sequences were aligned with MAFFT using default parameters (gap open penalty 1.53, gap extension penalty 0.123) (Katoh and Standley, 2013), and alignments (**Supplementary Data Sheets 3**, **4**) were subjected to maximum likelihood analysis with RAxML (Stamatakis, 2006) in each case employing 1000 bootstrap replicates (GTRCAT substitution model).

### Culture isolation

If available, a portion of the lung tissue was used in culturing on a yeast glucose media (1% yeast extract, 2% glucose, 1.5% agar) with the addition of tetracycline (10 mg/L) and chloramphenicol (50 mg/L). Typically, 3-4 small lung fragments (approximately 0.25 cm each) were plated onto a single 10-cm agar plate. Plates were incubated at 25°C until fungal growth was visible. Colonies arising from these segments were transferred as hyphal tips to fresh individual plates. Colony and microscopic characteristics were assessed before selecting isolates for ITS Sanger sequencing. After 2-7 days growth, tissue was collected, and DNA was extracted using the above CTAB procedure with the reduction of the heating step to 30 min at 65°C. PCR was performed as above with primers ITS1-F and ITS4 (White et al., 1990; Gardes and Bruns, 1993) primers followed by confirmation with gel electrophoresis confirmation. Products were purified with ExoSAP-IT (Affymetrix, Santa Clara, California) according to the manufacturer’s recommendations. The entire ITS region was targeted for Sanger sequencing using BigDye Terminator v3.1 (Applied Biosystems, Foster City, CA) and sequences were identified using BLAST (Altschul et al., 1990). Sequences from lung fungal cultures were deposited in GenBank under accession numbers MW652389-MW652417 (**Supplementary Table 4**).

## Data availability statement

The datasets presented in this study are available in online repositories. Access to the datasets is at https://www.ncbi.nlm.nih.gov/ with the accession number: PRJNA769405. Further inquiries should be directed to the corresponding author.

## Ethics statement

Ethical review and approval was not required for the animal study because this study involved frozen lung tissues from museum collections deposited over several decades by multiple researchers. No live animals were employed or sacrificed for the study. Although not directly related to our study, typical animal handling and current Animal Welfare Assurance protocols employed by scientists at the University of New Mexico Museum of Southwestern Biology are presented in Materials and Methods under the subheading “Tissue acquisition.”

## Author contributions

PS-H, DN, JT, and JC conceptualized the project. PS-H, KM, LM, SL, and JC collected data. PS-H, KC, SL, and DN analyzed the data. PS-H and DN prepared the initial draft of the manuscript, and all authors participated in revisions. PS-H, JT, and DN provided funding. All authors contributed to the article and approved the submitted version.

## Funding

PS-H was supported in part by a UNM Sevilleta LTER Summer Graduate Student Fellowship (NSF awards DEB 1655499 and DEB 1440478) and the UNM Sevilleta Field Station endowment fund, with additional support from research awards from the UNM Graduate and Professional Student Association and the Department of Biology Graduate Research Allocations Committee. PS-H was also supported by the Mycological Society of America Graduate Research Fellowship and the John W. Rippon Research Award. JT acknowledges the Valley Fever Research Initiative at the University of California, VFR-19-633952 and grant R01 AI148336 from the NIAID. JC acknowledges NSF support (NSF1561342, 2033482). Support was also provided by the UNM Department of Biology’s Molecular Biology Facility, supported by the UNM Center for Evolutionary & Theoretical Immunology (CETI) under National Institutes of Health grant P30GM110907.

## Acknowledgments

This project was made possible by frozen tissue collections preserved at the Museum of Southwestern Biology at the University of New Mexico (UNM) and the Museum of Vertebrate Zoology at University of California Berkeley. The authors would like to thank the field crews that collected these tissues, in particular Dr. James L. Patton. We additionally would like to thank the UNM Center for Advanced Research Computing, supported in part by the National Science Foundation (NSF), for providing high performance computing resources used in this work.

## Conflict of interest

The authors declare that the research was conducted in the absence of any commercial or financial relationships that could be construed as a potential conflict of interest.

## Publisher’s note

All claims expressed in this article are solely those of the authors and do not necessarily represent those of their affiliated organizations, or those of the publisher, the editors and the reviewers. Any product that may be evaluated in this article, or claim that may be made by its manufacturer, is not guaranteed or endorsed by the publisher.

## Supplementary material

Supplementary Data Sheet 1 Supplementary Figures and Tables.

Supplementary Data Sheet 2 ITS2 OTU frequency and abundance, including taxonomic classifications designated by UNITE database v8.2 (Nilsson et al., 2019) and BLAST searches.

Supplementary Data Sheet 3 Onygenales sequence alignment (NEXUS format).

Supplementary Data Sheet 4 Pneumocystidales sequence alignment (NEXUS format).
