# Supplementary material for: Breathing can be dangerous: Opportunistic fungal pathogens and the diverse community of the small mammal lung mycobiome
Source: Front Fungal Biol. 2022 Sep 26;3:996574. doi: 10.3389/ffunb.2022.996574 (PMC10512277; doi:10.3389/ffunb.2022.996574)
Supplement: Supplementary Data Sheet 1 — Supplementary Figures and Tables. [file DataSheet_1.pdf]

Supplementary Information

Breathing can be dangerous: Opportunistic fungal pathogens and the diverse community of the lung mycobiome

Paris S. Salazar-Hamm, Kyana N. Montoya, Liliam Montoya, Kel Cook, Schuyler Liphardt, John W. Taylor, Joseph A. Cook, and Donald O. Natvig

Correspondence:

Paris S. Salazar-Hamm
Email: psh102@unm.edu

Table of Contents

Supplementary Figures 1 to 5 2-6
Supplementary Tables 1 to 5 7-16

Not included in this PDF:

Supplementary Datasets 1 to 3

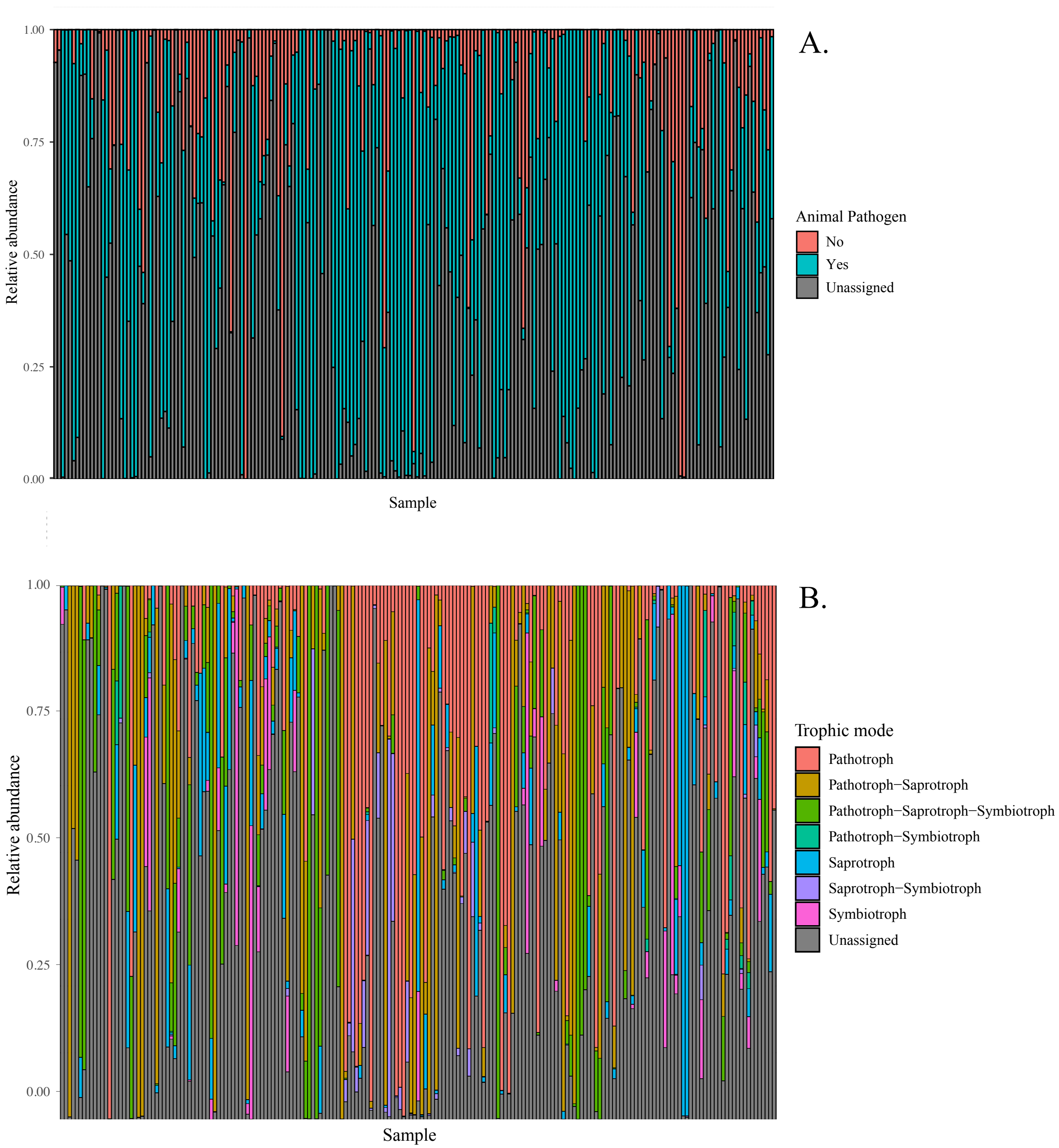

Supplementary Figure 1. Relative abundance of fungal trophic modes (A) and pooled animal pathogen fungal guilds (B) using FUNGuild (Nguyen et al. 2016) for fungal OTU assignments. Each bar along the x-axis represents one sample.

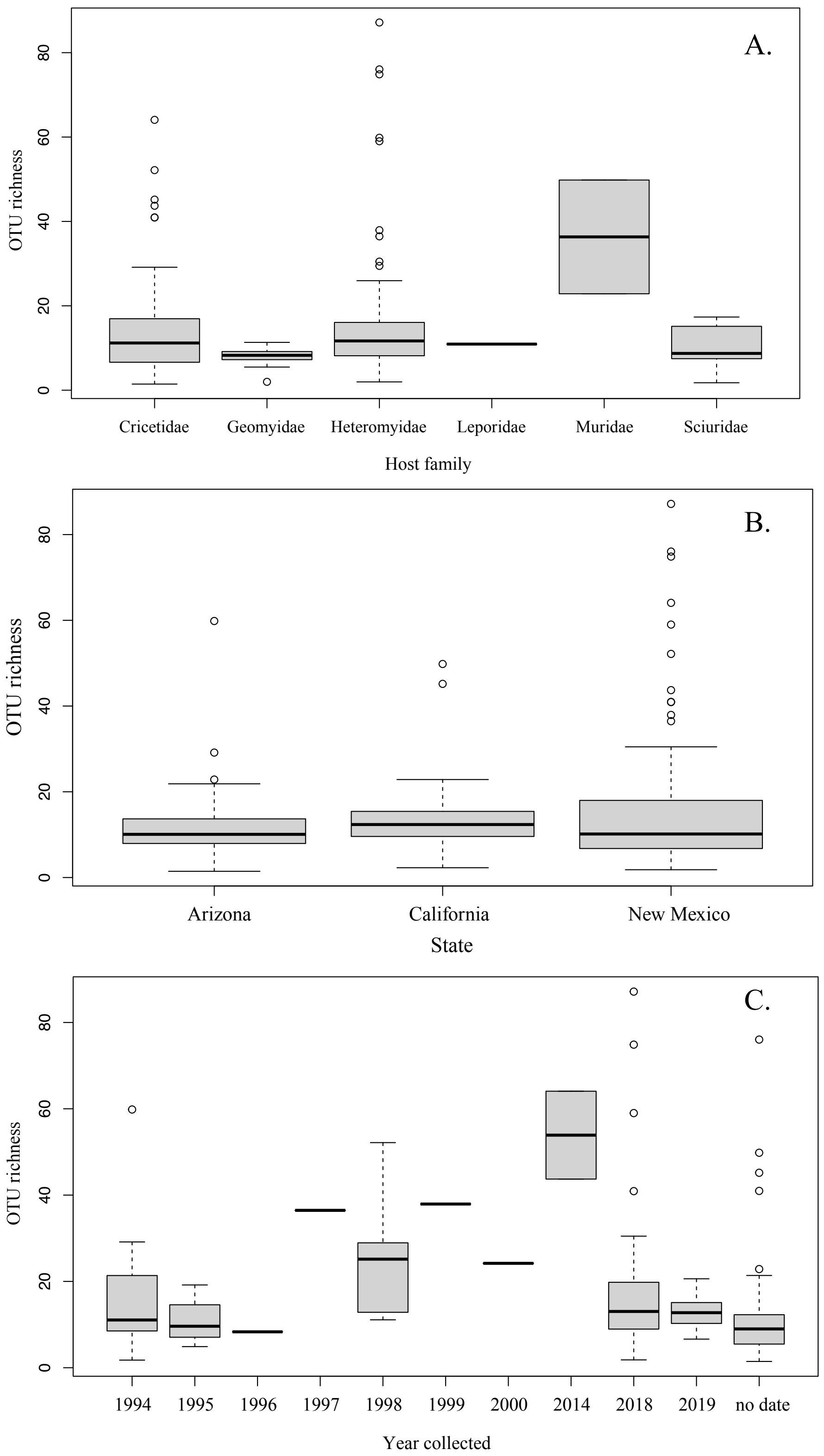

Supplementary Figure 2. Alpha diversity calculated by OTU richness showed no statistically significant differences among host family (A), state (B), or collection year (C). We note, however, that sample sizes differed by family, state, and collection year.

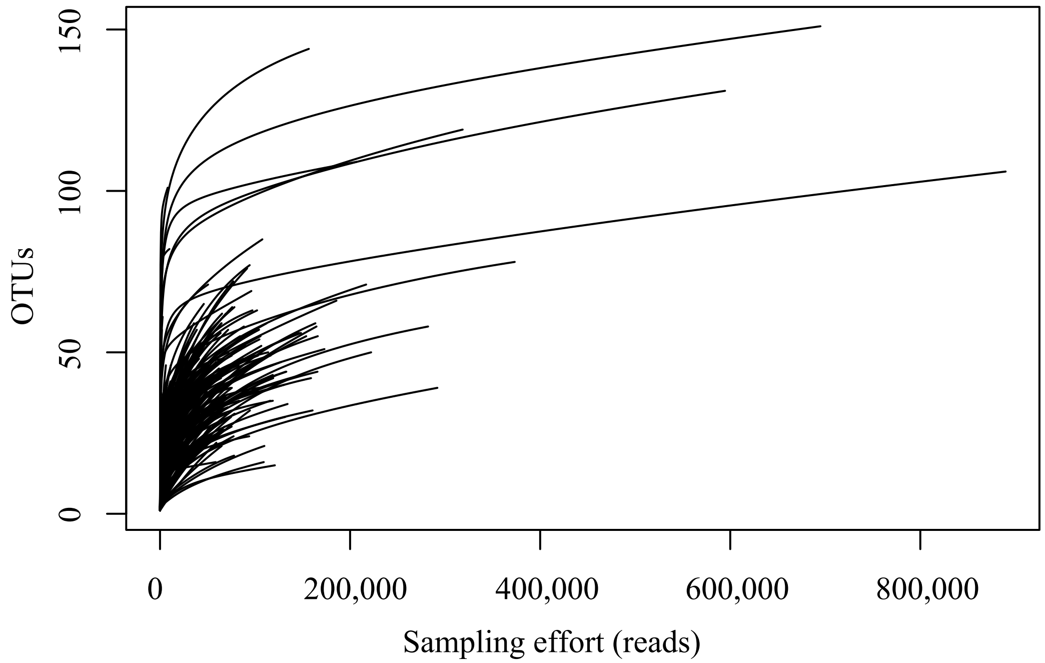

Supplementary Figure 3. Rarefaction curves for fungal OTU richness given sampling effort for 199 small mammalian lung tissues.

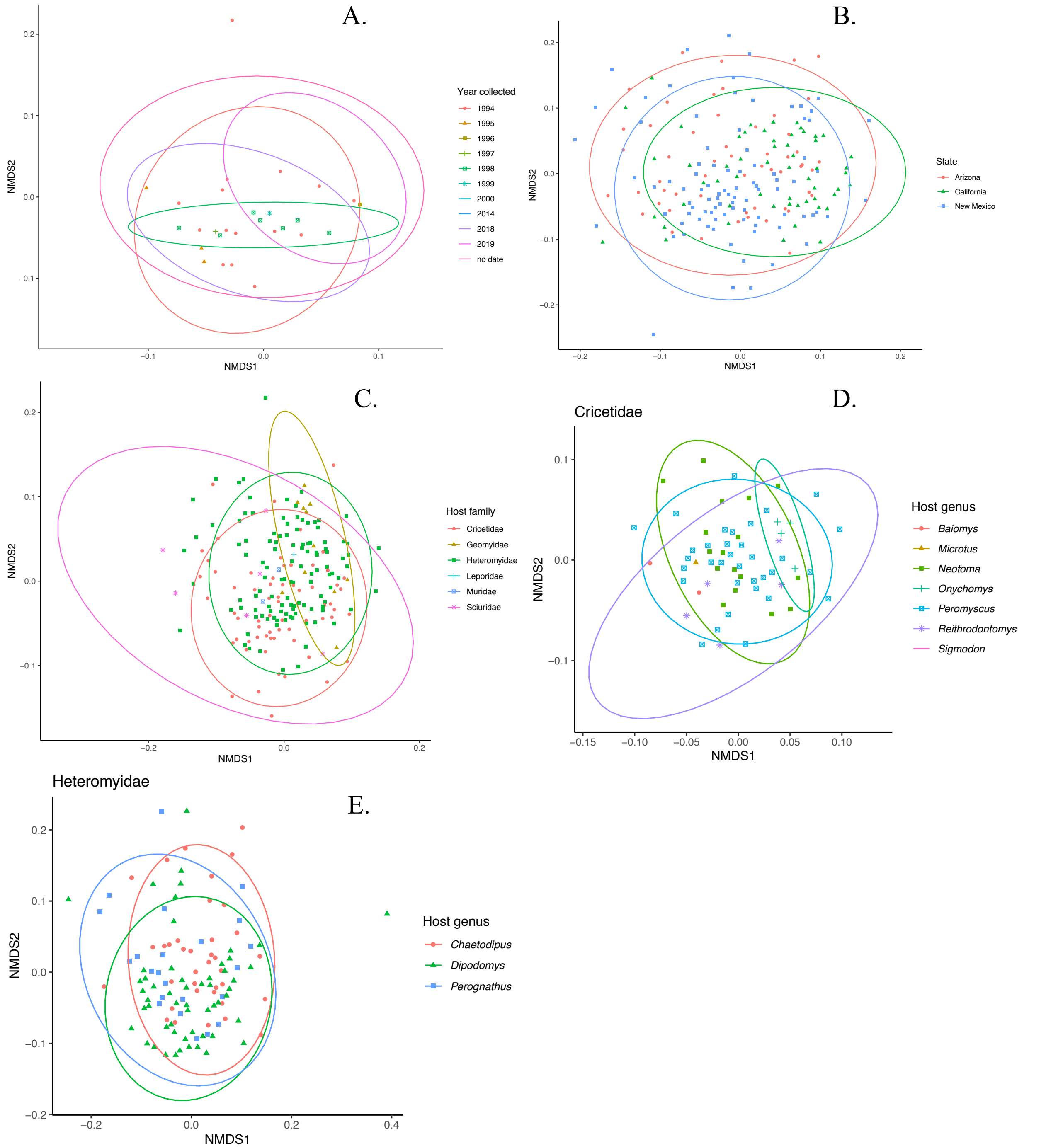

Supplementary Figure 4. NMDS ordinations of small-mammal samples were utilized to visualize lung mycobiome composition among small mammals across time (year collected, A), space (state, B), and host family (C). Examination of deeply sampled host families failed to demonstrate differences in fungal community by host genus within either the Cricetidae (D) or Heteromyidae (E).

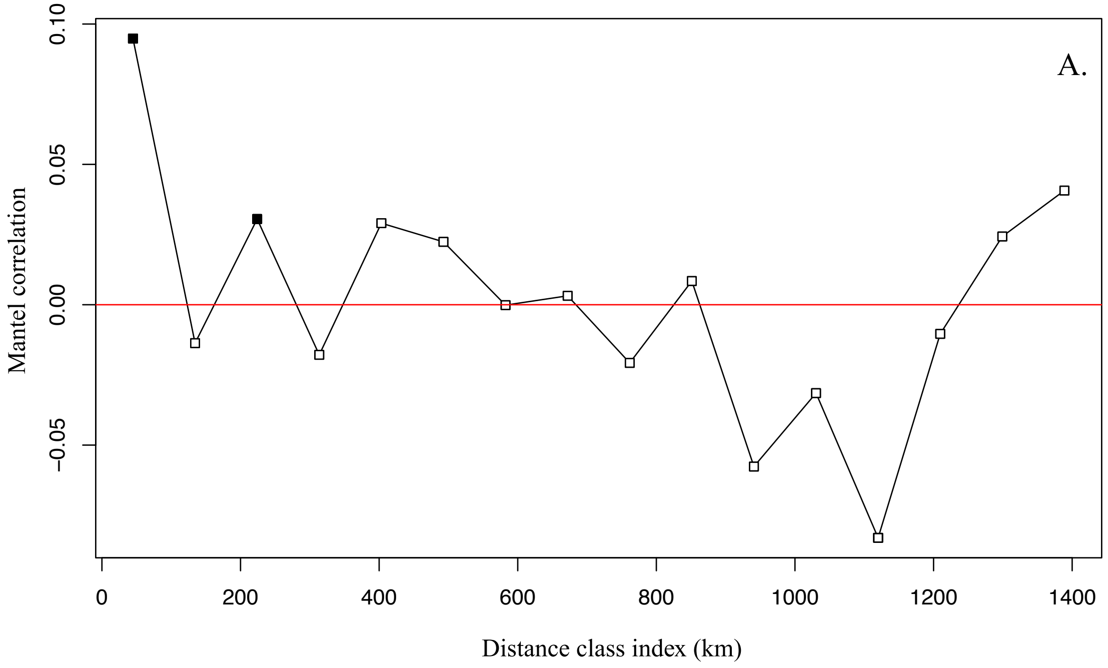

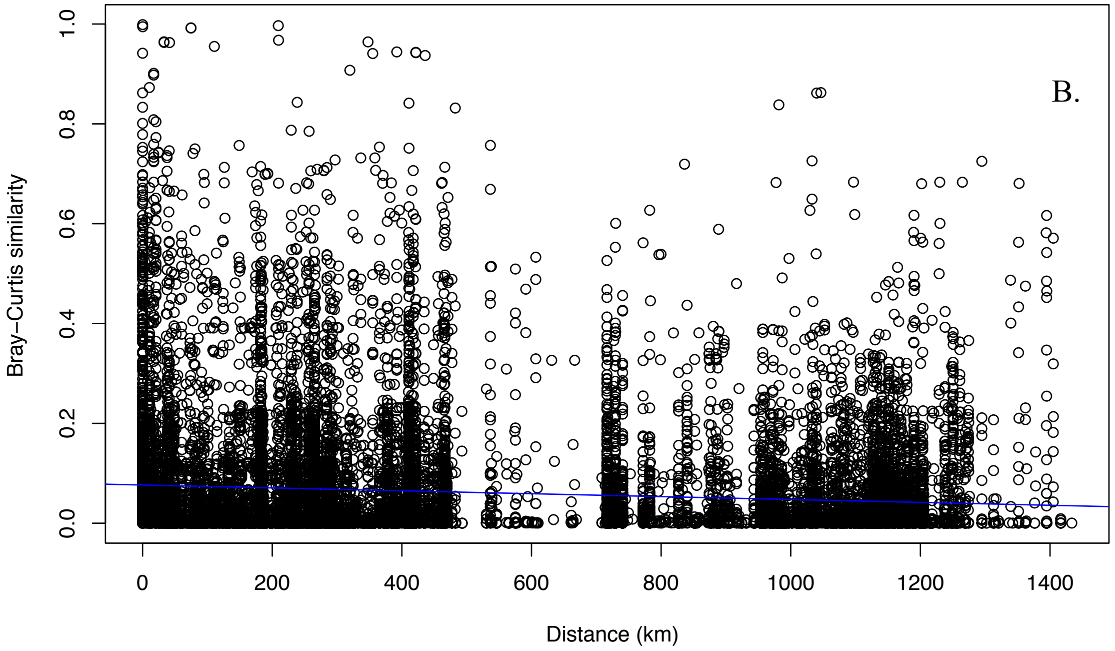

Supplementary Figure 5. Spatial structure was assessed using a Mantel correlogram (A) and distance decay plot (B). (A) The Mantel test revealed significant positive correlation between geographic distance and fungal community dissimilarity (r=0.1132, p=0.001). Positive spatial autocorrelation was seen at two distance classes, less than 44.8 km and between 135 km and 224 km (solid black boxes). (B) While fungal communities in general exhibited low similarity among samples, distance decay analysis indicated decreasing similarity along a geographical scale (p<0.001). The line of best fit is shown in blue.

Supplementary Table 1. Samples from which frozen tissues were obtained for Illumina ITS2 sequencing. Host identification of museum specimens was confirmed by cyt b mitochondrial sequencing and examination of voucher specimens.

Voucher	Host ID	Locality	Cyt b GenBank Accession Num.
MVZ:Mamm:239384	*Dipodomys heermanni*	Kern Co. CA	OK135085
MVZ:Mamm:239385	*Dipodomys heermanni*	Kern Co. CA	OK135058
MVZ:Mamm:239386	*Dipodomys heermanni*	Kern Co. CA	OK135089
MVZ:Mamm:239387	*Dipodomys heermanni*	Kern Co. CA	OK135087
MVZ:Mamm:239388	*Dipodomys heermanni*	Kern Co. CA	OK135057
MVZ:Mamm:239389	*Dipodomys heermanni*	Kern Co. CA	OK135054
MVZ:Mamm:239390	*Dipodomys heermanni*	Kern Co. CA	OK135059
MVZ:Mamm:239391	*Dipodomys heermanni*	Kern Co. CA	OK135082
MVZ:Mamm:239392	*Dipodomys heermanni*	Kern Co. CA	OK135065
MVZ:Mamm:239393	*Dipodomys heermanni*	Kern Co. CA	OK135060
MVZ:Mamm:239394	*Dipodomys heermanni*	Kern Co. CA	OK135104
MVZ:Mamm:239395	*Dipodomys heermanni*	Kern Co. CA	OK135119
MVZ:Mamm:239396	*Dipodomys heermanni*	Kern Co. CA	OK135105
MVZ:Mamm:239397	*Dipodomys heermanni*	Kern Co. CA	OK135072
MVZ:Mamm:239409	*Dipodomys nitratoides*	Kern Co. CA	-
MVZ:Mamm:239410	*Dipodomys nitratoides*	Kern Co. CA	-
MVZ:Mamm:239411	*Dipodomys nitratoides*	Kern Co. CA	-
MVZ:Mamm:239412	*Dipodomys nitratoides*	Kern Co. CA	-
MVZ:Mamm:239413	*Dipodomys nitratoides*	Kern Co. CA	-
MVZ:Mamm:239414	*Dipodomys nitratoides*	Kern Co. CA	OK135078
MVZ:Mamm:239415	*Dipodomys nitratoides*	Kern Co. CA	-
MVZ:Mamm:239416	*Dipodomys nitratoides*	Kern Co. CA	-
MVZ:Mamm:239418	*Dipodomys nitratoides*	Kern Co. CA	-
MVZ:Mamm:239419	*Dipodomys nitratoides*	Kern Co. CA	-
MVZ:Mamm:239505	*Perognathus inornatus*	Kern Co. CA	OK135093
MVZ:Mamm:239506	*Perognathus inornatus*	Kern Co. CA	OK135066
MVZ:Mamm:239507	*Perognathus inornatus*	Kern Co. CA	-
MVZ:Mamm:239508	*Perognathus inornatus*	Kern Co. CA	-
MVZ:Mamm:239509	*Perognathus inornatus*	Kern Co. CA	OK135074
MVZ:Mamm:239510	*Perognathus inornatus*	Kern Co. CA	OK135083
MVZ:Mamm:239511	*Perognathus inornatus*	Kern Co. CA	-
MVZ:Mamm:239512	*Perognathus inornatus*	Kern Co. CA	OK135079
MVZ:Mamm:239597	*Onychomys torridus*	Kern Co. CA	OK134994
MVZ:Mamm:239598	*Onychomys torridus*	Kern Co. CA	OK135090
MVZ:Mamm:239599	*Onychomys torridus*	Kern Co. CA	OK135012

MVZ:Mamm:239619	*Peromyscus maniculatus*	Kern Co. CA	OK134996
MVZ:Mamm:239621	*Peromyscus maniculatus*	Kern Co. CA	OK134997
MVZ:Mamm:239622	*Peromyscus maniculatus*	Kern Co. CA	OK134998
MVZ:Mamm:239623	*Peromyscus maniculatus*	Kern Co. CA	OK134999
MVZ:Mamm:239641	*Reithrodontomys megalotis*	Kern Co. CA	OK135000
MSB:Mamm:72326	*Perognathus flavescens*	Socorro Co. NM	OK135028
MSB:Mamm:72516	*Dipodomys merriami*	Socorro Co. NM	OK135120
MSB:Mamm:72525	*Dipodomys merriami*	Socorro Co. NM	OK135029
MSB:Mamm:72555	*Peromyscus eremicus*	Socorro Co. NM	OK135001
MSB:Mamm:73101	*Chaetodipus baileyi*	Maricopa Co. AZ	OK135115
MSB:Mamm:73109	*Chaetodipus baileyi*	Maricopa Co. AZ	OK135113
MSB:Mamm:73113	*Chaetodipus baileyi*	Maricopa Co. AZ	OK135073
MSB:Mamm:73114	*Sigmodon arizonae*	Maricopa Co. AZ	OK135052
MSB:Mamm:75330	*Onychomys torridus*	Maricopa Co. AZ	OK135062
MSB:Mamm:75338	*Peromyscus eremicus*	Maricopa Co. AZ	OK135002
MSB:Mamm:75343	*Neotoma albigula*	Maricopa Co. AZ	OK135003
77240 (no voucher)	*Dipodomys merriami*	Maricopa Co. AZ	OK135098
MSB:Mamm:77241	*Ammospermophilus harrisii*	Maricopa Co. AZ	OK135018
MSB:Mamm:77244	*Dipodomys merriami*	Maricopa Co. AZ	OK135041
MSB:Mamm:77246	*Dipodomys merriami*	Maricopa Co. AZ	OK135053
MSB:Mamm:77247	*Dipodomys merriami*	Maricopa Co. AZ	OK135108
MSB:Mamm:77250	*Dipodomys merriami*	Maricopa Co. AZ	OK135112
77253 (no voucher)	*Dipodomys heermanni*	Maricopa Co. AZ	OK135135
77314 (no voucher)	*Sigmodon arizonae*	Maricopa Co. AZ	OK135100
MSB:Mamm:77316	*Perognathus amplus*	Maricopa Co. AZ	OK135136
MSB:Mamm:77317	*Perognathus amplus*	Maricopa Co. AZ	OK135081
MSB:Mamm:77318	*Perognathus amplus*	Maricopa Co. AZ	-
MSB:Mamm:77325	*Chaetodipus penicillatus*	Maricopa Co. AZ	OK135086
MSB:Mamm:77339	*Neotoma albigula*	Maricopa Co. AZ	OK135013
MSB:Mamm:77340	*Neotoma albigula*	Maricopa Co. AZ	OK135006
MSB:Mamm:77680	*Reithrodontomys megalotis*	Cochise Co. AZ	OK135016
MSB:Mamm:77687	*Baiomys taylori*	Cochise Co. AZ	OK135004
MSB:Mamm:77688	*Reithrodontomys megalotis*	Cochise Co. AZ	OK135020
MSB:Mamm:77712	*Chaetodipus penicillatus*	Cochise Co. AZ	OK135069
MSB:Mamm:77713	*Chaetodipus penicillatus*	Cochise Co. AZ	OK135042
MSB:Mamm:77717	*Chaetodipus penicillatus*	Cochise Co. AZ	OK135070
MSB:Mamm:77719	*Chaetodipus penicillatus*	Cochise Co. AZ	OK135043
MSB:Mamm:77721	*Chaetodipus penicillatus*	Cochise Co. AZ	OK135051
MSB:Mamm:77725	*Dipodomys merriami*	Cochise Co. AZ	-

MSB:Mamm:77726	*Dipodomys merriami*	Cochise Co. AZ	-
MSB:Mamm:77727	*Dipodomys ordii*	Cochise Co. AZ	OK135025
MSB:Mamm:77728	*Dipodomys ordii*	Cochise Co. AZ	OK135122
MSB:Mamm:78077	*Sylvilagus audubonii*	Cochise Co. AZ	OK135033
MSB:Mamm:78084	*Peromyscus leucopus*	Cochise Co. AZ	OK135005
MSB:Mamm:78106	*Chaetodipus penicillatus*	Cochise Co. AZ	OK135023
MSB:Mamm:78110	*Dipodomys merriami*	Cochise Co. AZ	OK135030
MSB:Mamm:78111	*Dipodomys merriami*	Cochise Co. AZ	OK135133
MSB:Mamm:78112	*Dipodomys merriami*	Cochise Co. AZ	-
78118 (no voucher)	*Ammospermophilus harrisii*	Cochise Co. AZ	OK135099
MSB:Mamm:78136	*Neotoma albigula*	Cochise Co. AZ	OK135132
MSB:Mamm:78217	*Chaetodipus baileyi*	Pima Co. AZ	OK135061
MSB:Mamm:79466	*Dipodomys merriami*	Socorro Co. NM	OK135071
MSB:Mamm:79868	*Neotoma albigula*	Socorro Co. NM	OK135019
MSB:Mamm:81689	*Peromyscus maniculatus*	Kings Co. CA	OK135128
MSB:Mamm:81700	*Peromyscus maniculatus*	Kings Co. CA	-
MSB:Mamm:81704	*Peromyscus maniculatus*	Kings Co. CA	OK135007
MSB:Mamm:81711	*Mus musculus*	Kings Co. CA	OK135123
MSB:Mamm:81715	*Mus musculus*	Kings Co. CA	OK135114
MSB:Mamm:83441	*Chaetodipus baileyi*	Pima Co. AZ	OK135044
MSB:Mamm:84453	*Peromyscus maniculatus*	Tulare Co. CA	OK135141
MSB:Mamm:84466	*Perognathus parvus*	Mono Co. CA	OK135126
MSB:Mamm:86216	*Peromyscus maniculatus*	Lassen Co. CA	OK135008
MSB:Mamm:87435	*Peromyscus crinitus*	San Bernardino Co. CA	OK135015
MSB:Mamm:87516	*Perognathus longimembris*	San Bernardino Co. CA	OK135134
MSB:Mamm:87605	*Reithrodontomys fulvescens*	Cochise Co. AZ	-
MSB:Mamm:87794	*Neotoma albigula*	Socorro Co. NM	OK135129
MSB:Mamm:87863	*Dipodomys ordii*	Catron Co. NM	OK135055
MSB:Mamm:87873	*Microtus mogollonensis*	Cibola Co. NM	OK135031
MSB:Mamm:87948	*Dipodomys ordii*	Cibola Co. NM	OK135124
MSB:Mamm:87949	*Dipodomys ordii*	Cibola Co. NM	OK135138
MSB:Mamm:88099	*Perognathus flavus*	Catron Co. NM	OK135088
MSB:Mamm:89733	*Dipodomys ordii*	Socorro Co. NM	OK135045
MSB:Mamm:89748	*Dipodomys ordii*	Socorro Co. NM	OK135121
MSB:Mamm:89752	*Neotoma albigula*	Socorro Co. NM	OK135009
89766 (no voucher)	*Perognathus flavus*	Socorro Co. NM	OK135142
MSB:Mamm:89796	*Dipodomys ordii*	Socorro Co. NM	OK135106
MSB:Mamm:90911	*Dipodomys merriami*	Socorro Co. NM	OK135116
MSB:Mamm:90965	*Perognathus flavus*	Socorro Co. NM	OK135022

91253 (no voucher)	*Perognathus flavus*	Socorro Co. NM	OK135137
MSB:Mamm:91260	*Peromyscus californicus*	Monterey Co. CA	-
MSB:Mamm:91271	*Peromyscus californicus*	Monterey Co. CA	-
MSB:Mamm:91400	*Chaetodipus californicus*	Obispo Co. CA	OK135021
MSB:Mamm:91401	*Chaetodipus californicus*	Obispo Co. CA	OK135046
MSB:Mamm:93037	*Perognathus flavus*	McKinley Co. NM	OK135047
MSB:Mamm:96300	*Dipodomys ordii*	McKinley Co. NM	-
MSB:Mamm:139034	*Dipodomys ordii*	McKinley Co. NM	OK135118
MSB:Mamm:146153	*Perognathus flavus*	Cochise Co. AZ	OK135101
MSB:Mamm:146226	*Chaetodipus baileyi*	Cochise Co. AZ	OK135102
MSB:Mamm:150582	*Thomomys bottae*	San Juan Co. NM	-
MSB:Mamm:150583	*Thomomys bottae*	San Juan Co. NM	-
MSB:Mamm:150584	*Thomomys bottae*	San Juan Co. NM	-
MSB:Mamm:156712	*Dipodomys merriami*	Grant Co. NM	OK135107
MSB:Mamm:199648	*Chaetodipus intermedius*	Catron Co. NM	OK135130
MSB:Mamm:231069	*Thomomys bottae*	San Juan Co. NM	-
MSB:Mamm:239509	*Peromyscus leucopus*	Cochise Co. AZ	OK134972
MSB:Mamm:244556	*Peromyscus boylii*	Pima Co. AZ	OK134973
MSB:Mamm:244564	*Peromyscus boylii*	Pima Co. AZ	OK134974
MSB:Mamm:244584	*Chaetodipus baileyi*	Pima Co. AZ	OK135131
MSB:Mamm:244630	*Chaetodipus baileyi*	Pima Co. AZ	OK135103
244631 (no voucher)	*Peromyscus boylii*	Pima Co. AZ	OK135096
MSB:Mamm:253299	*Reithrodontomys montanus*	Socorro Co. NM	OK134975
MSB:Mamm:259869	*Sigmodon hispidus*	Socorro Co. NM	OK135048
MSB:Mamm:262655	*Chaetodipus intermedius*	Grant Co. NM	OK135034
MSB:Mamm:263654	*Dipodomys merriami*	Sierra Co. NM	-
MSB:Mamm:263880	*Dipodomys ordii*	Grant Co. NM	-
MSB:Mamm:269016	*Perognathus merriami*	Hidalgo Co. NM	OK135035
MSB:Mamm:269115	*Thomomys bottae*	Sierra Co. NM	-
MSB:Mamm:269217	*Thomomys bottae*	Sierra Co. NM	-
MSB:Mamm:270443	*Peromyscus boylii*	Sierra Co. NM	OK134976
MSB:Mamm:270470	*Neotoma albigula*	Sierra Co. NM	OK257677
MSB:Mamm:279264	*Thomomys bottae*	Sierra Co. NM	-
MSB:Mamm:281843	*Chaetodipus formosus*	San Bernardino Co. CA	OK135084
MSB:Mamm:282890	*Peromyscus maniculatus*	Cochise Co. AZ	OK134977
MSB:Mamm:284485	*Thomomys bottae*	Catron Co. NM	-
MSB:Mamm:291392	*Dipodomys merriami*	Grant Co. NM	-
MSB:Mamm:291445	*Peromyscus boylii*	Grant Co. NM	OK135011
MSB:Mamm:303043	*Neotoma stephensi*	Sierra Co. NM	-

MSB:Mamm:303044	*Neotoma albigula*	Sierra Co. NM	OK135067
MSB:Mamm:303045	*Neotoma albigula*	Sierra Co. NM	OK135127
MSB:Mamm:303046	*Otospermophilus variegatus*	Sierra Co. NM	OK135032
MSB:Mamm:303047	*Neotoma stephensi*	Sierra Co. NM	OK134978
MSB:Mamm:303049	*Chaetodipus intermedius*	Sierra Co. NM	OK135068
MSB:Mamm:304173	*Chaetodipus intermedius*	Sierra Co. NM	OK135036
MSB:Mamm:304174	*Chaetodipus intermedius*	Sierra Co. NM	OK135037
MSB:Mamm:304175	*Chaetodipus intermedius*	Sierra Co. NM	OK135038
MSB:Mamm:304176	*Chaetodipus intermedius*	Sierra Co. NM	OK135024
MSB:Mamm:304177	*Tamias dorsalis*	Sierra Co. NM	OK135125
MSB:Mamm:304178	*Neotoma albigula*	Sierra Co. NM	-
MSB:Mamm:304180	*Peromyscus boylii*	Sierra Co. NM	OK134980
MSB:Mamm:304181	*Peromyscus boylii*	Sierra Co. NM	OK134981
MSB:Mamm:304183	*Peromyscus boylii*	Sierra Co. NM	OK134983
MSB:Mamm:304184	*Peromyscus boylii*	Sierra Co. NM	OK134984
MSB:Mamm:304185	*Peromyscus boylii*	Sierra Co. NM	-
MSB:Mamm:304186	*Peromyscus boylii*	Sierra Co. NM	OK134985
MSB:Mamm:304190	*Peromyscus boylii*	Sierra Co. NM	OK134989
MSB:Mamm:304191	*Peromyscus boylii*	Sierra Co. NM	OK134990
MSB:Mamm:304192	*Peromyscus boylii*	Sierra Co. NM	OK134991
MSB:Mamm:305233	*Thomomys bottae*	Sierra Co. NM	-
MSB:Mamm:305235	*Neotoma albigula*	Sierra Co. NM	-
MSB:Mamm:305236	*Neotoma albigula*	Sierra Co. NM	-
MSB:Mamm:305237	*Otospermophilus variegatus*	Sierra Co. NM	OK135139
MSB:Mamm:305238	*Neotoma albigula*	Sierra Co. NM	-
MSB:Mamm:305240	*Chaetodipus intermedius*	Sierra Co. NM	OK135026
MSB:Mamm:305241	*Dipodomys merriami*	Sierra Co. NM	OK135027
MSB:Mamm:305242	*Chaetodipus intermedius*	Sierra Co. NM	OK135094
MSB:Mamm:305243	*Neotoma albigula*	Sierra Co. NM	OK135017
MSB:Mamm:305244	*Dipodomys merriami*	Sierra Co. NM	-
MSB:Mamm:305245	*Chaetodipus intermedius*	Sierra Co. NM	OK135039
305246 (no voucher)	*Otospermophilus variegatus*	Sierra Co. NM	OK135097
MSB:Mamm:305247	*Chaetodipus intermedius*	Sierra Co. NM	OK135056
MSB:Mamm:305257	*Peromyscus boylii*	Sierra Co. NM	OK135014
MSB:Mamm:305258	*Peromyscus boylii*	Sierra Co. NM	OK134992
MSB:Mamm:305259	*Peromyscus boylii*	Sierra Co. NM	OK134993
MSB:Mamm:323706	*Dipodomys merriami*	Luna Co. NM	OK135092
MSB:Mamm:323714	*Chaetodipus intermedius*	Luna Co. NM	OK135140
MSB:Mamm:323716	*Chaetodipus intermedius*	Luna Co. NM	OK135040

MSB:Mamm:323717	*Chaetodipus intermedius*	Luna Co. NM	OK135080
MSB:Mamm:323718	*Chaetodipus intermedius*	Luna Co. NM	OK135049
MSB:Mamm:323732	*Dipodomys merriami*	Luna Co. NM	OK135110
MSB:Mamm:323735	*Chaetodipus intermedius*	Luna Co. NM	OK135064
MSB:Mamm:324781	*Perognathus flavus*	Catron Co. NM	OK135050
MSB:Mamm:325306	*Thomomys bottae*	Catron Co. NM	-
MSB:Mamm:325312	*Peromyscus truei*	Catron Co. NM	-
MSB:Mamm:325314	*Thomomys bottae*	Catron Co. NM	-

Supplementary Table 2. ***Coccidioides*** **positive samples based on Illumina sequencing of the fungal ribosomal ITS2 region.**

**Host Voucher**	**Host ID**	**County**	**State**	**OTU 136**	**OTU 899**	**Total reads (Rel. abundance)**
78118 (no voucher)	*Ammospermophilus harrisii*	Cochise	AZ	49	4	53 (<1%)
MSB:Mamm:78106	*Chaetodipus penicillatus*	Cochise	AZ	36	0	36 (<1%)
MSB:Mamm:78110	*Dipodomys merriami*	Cochise	AZ	1692	182	1874 (<1%)
MSB:Mamm:78077	*Sylvilagus audubonii*	Cochise	AZ	855	128	983 (1.6%)
MSB:Mamm:77713	*Chaetodipus penicillatus*	Maricopa	AZ	186	15	102 (<1%)
MSB:Mamm:77719	*Chaetodipus penicillatus*	Maricopa	AZ	1974	2154	4128 (6.3%)
77240 (no voucher)	*Dipodomys merriami*	Maricopa	AZ	2137	0	2137 (2.8%)
77253 (no voucher)	*Dipodomys heermanni*	Maricopa	AZ	1	0	1 (<1%)
MSB:Mamm:77316	*Perognathus amplus*	Maricopa	AZ	0	1	1 (<1%)
MVZ:Mamm:239395	*Dipodomys heermanni*	Kern	CA	0	1	1 (<1%)
MVZ:Mamm:239414	*Dipodomys nitratoides*	Kern	CA	6	0	6 (<1%)
MVZ:Mamm:239597	*Onychomys torridus*	Kern	CA	19	8	27 (<1%)
MVZ:Mamm:239619	*Peromyscus maniculatus*	Kern	CA	2	0	2 (<1%)
MSB:Mamm:284485	*Thomomys bottae*	Catron	NM	1	1	2 (<1%)
MSB:Mamm:304176	*Chaetodipus intermedius*	Sierra	NM	1	0	1 (<1%)
MSB:Mamm:305240	*Chaetodipus intermedius*	Sierra	NM	4410	21	4431 (1.4%)
MSB:Mamm:305241	*Dipodomys merriami*	Sierra	NM	2972	2	2974 (<1%)
MSB:Mamm:305238	*Neotoma albigula*	Sierra	NM	129	0	129 (10.6%)
MSB:Mamm:305243	*Neotoma albigula*	Sierra	NM	765	239	1004 (1%)
MSB:Mamm:303043	*Neotoma stephensi*	Sierra	NM	130	0	130 (<1%)
305246 (no voucher)	*Otospermophilus variegatus*	Sierra	NM	3	0	3 (<1%)
MSB:Mamm:270443	*Peromyscus boylii*	Sierra	NM	123	74	197 (<1%)
MSB:Mamm:304190	*Peromyscus boylii*	Sierra	NM	1	0	1 (<1%)
MSB:Mamm:89752	*Neotoma albigula*	Socorro	NM	25	36	61 (<1%)

Supplementary Table 3. Spearman correlations at different rarefaction depths of Illumina ITS2 sequences demonstrate no significant correlation between *Blastomyces parvus* OTUs and *Coccidioides* OTUs.

Rarefied Depth	Samples	Rarefied OTUs	Spearman correlation	p-value
1000	199	642	0.01773099	0.387
5000	189	577	-0.008329599	0.5438
10000	182	531	-0.0257075	0.6351

Supplementary Table 4. Fungi isolated from small rodent lungs in Kern County California.

Organism	GenBank	Host ID	Voucher
Blastomyces parvus	MW652389	*Dipodomys heermanni*	MVZ:Mamm:239395
Blastomyces parvus	MW652390	*Perognathus inornatus*	MVZ:Mamm:239512
Aspergillus fumigatus	MW652391	*Dipodomys heermanni*	MVZ:Mamm:239386
Aspergillus sp.	MW652392	*Perognathus inornatus*	MVZ:Mamm:239508
Circinella sp.	MW652393	*Dipodomys nitratoides*	MVZ:Mamm:239418
Circinella sp.	MW652394	*Dipodomys nitratoides*	MVZ:Mamm:239418
Circinella sp.	MW652395	*Onychomys torridus*	MVZ:Mamm:239598
Circinella sp.	MW652396	*Perognathus inornatus*	MVZ:Mamm:239505
Circinella sp.	MW652397	*Perognathus inornatus*	MVZ:Mamm:239506
Subramaniula cuniculorum	MW652398	*Peromyscus maniculatus*	MVZ:Mamm:239618
Chaetomium sp.	MW652399	*Peromyscus maniculatus*	MVZ:Mamm:239622
Blastomyces parvus	MW652400	*Dipodomys nitratoides*	MVZ:Mamm:239411
Blastomyces parvus	MW652401	*Dipodomys nitratoides*	MVZ:Mamm:239416
Blastomyces parvus	MW652402	*Dipodomys heermanni*	MVZ:Mamm:239414
Blastomyces parvus	MW652403	*Dipodomys nitratoides*	MVZ:Mamm:239413
Blastomyces parvus	MW652404	*Dipodomys heermanni*	MVZ:Mamm:239396
Blastomyces parvus	MW652405	*Dipodomys heermanni*	MVZ:Mamm:239394
Blastomyces parvus	MW652406	*Dipodomys nitratoides*	MVZ:Mamm:239414
Lichtheimia ramosa	MW652407	*Dipodomys nitratoides*	MVZ:Mamm:239412
Penicillium sp.	MW652408	*Peromyscus maniculatus*	MVZ:Mamm:239620
Penicillium sp.	MW652409	*Onychomys torridus*	MVZ:Mamm:239599
Penicillium sp.	MW652410	*Dipodomys nitratoides*	MVZ:Mamm:239419
Penicillium sp.	MW652411	*Dipodomys nitratoides*	MVZ:Mamm:239419
Penicillium sp.	MW652412	*Dipodomys heermanni*	MVZ:Mamm:239388
Penicillium sp.	MW652413	*Perognathus inornatus*	MVZ:Mamm:239505
Penicillium sp.	MW652414	*Perognathus inornatus*	MVZ:Mamm:239512
Penicillium sp.	MW652415	*Dipodomys heermanni*	MVZ:Mamm:239387
Blastomyces parvus	MW652416	*Dipodomys heermanni*	MVZ:Mamm:239391
Emmonsiellopsis sp.	MW652417	*Dipodomys heermanni*	MVZ:Mamm:239397

Supplementary Table 5. BLAST searches of Eurotiomycetes OTUs demonstrate the lung environment is enriched for Onygenales over outdoor environments. Unlike OTUs from other Eurotiomycetes, OTUs from the Onygenales typically do not have close relatives among sequences from environmental samples in GenBank. Results are shown for the eight most frequent OTUs from the Onygenales and Eurotiales and their hits against the full nucleotide collection (nr/nt database) versus sequences limited to environmental surveys (uncultured fungi).

**Order**	**OTU**	**Top nr/nt hit (E value, % identity, organism)**	**Top hit limited to uncultured fungi (E value, % identity)**
Onygenales	OTU20	0.0, 97.28, *Blastomyces parvus*	2e-121, 94.43
	OTU106	0.0, 99.00, *Blastomyces parvus*	2e-131, 90.91
	OTU58	0.0, 96.29, *Blastomyces parvus*	1e-123, 89.59
	OTU1155	0.0, 96.80, *Blastomyces parvus*	1e-122, 89.37
	OTU15	0.0, 98.98 *Emmonsia crescens*	4e-128, 90.65
	OTU59	0.0, 99.17, *Auxarthron umbrinum*	1e-157, 97.83
	OTU85	0.0, 97.87, *Emmonsiellopsis* sp.	2e-151, 94.78
	OTU136	0.0, 99.21, *Coccidioides posadasii*	2e-131, 91.45
Eurotiales	OTU7	0.0, 99.21, *Penicillium mononematosum*	0.0, 98.68
	OTU3	0.0, 99.48, *Aspergillus* sp.	0.0, 99.48
	OTU11	0.0, 98.94, *Penicillium* sp.	0.0, 98.15
	OTU1118	0.0, 97.38, *Penicillium sp.*	0.0, 97.11
	OTU44	0.0, 99.22, *Thermomyces lanuginosus*	0.0, 99.22
	OTU75	0.0, 98.95, *Penicillium* sp.	0.0, 98.95
	OTU36	0.0, 99.48, *Aspergillus tubingensis*	0.0, 99.48
	OTU411	0.0, 99.47, *Penicillium* sp.	0.0, 98.94
